# Construction of Recombinant PRV Expressing PRRSV Antigens and Evaluation of Their Protective Efficacy in Pigs Against Homologous Challenge

**DOI:** 10.3390/vaccines14100915

**Published:** 2026-10-21

**Authors:** Shanghui Wang, Yunfei Liu, Longfei Han, Liguo Gao, Guangqiang Ye, Xiang Zhao, Yi Liu, Hongyang Liu, Zhaoxia Zhang, Changjiang Weng

**Affiliations:** 1 Division of Fundamental Immunology, National African Swine Fever Para-Reference Laboratory, State Key Laboratory for Animal Disease Control and Prevention, Harbin Veterinary Research Institute, Chinese Academy of Agricultural Sciences, Harbin 150069, China; 2 Heilongjiang Provincial Key Laboratory of Veterinary Immunology, Harbin 150069, China

**Keywords:** pseudorabies virus, highly pathogenic porcine reproductive and respiratory syndrome virus, live vector vaccine, CRISPR/Cas9, homologous recombination

## Abstract

**Background/Objectives:** Porcine reproductive and respiratory syndrome virus (PRRSV) remains a major threat to the global swine industry, while the emergence of variant pseudorabies virus (PRV) strains has further complicated disease control in China. This study aimed to develop a PRV-based viral vector platform for PRRSV antigen delivery and evaluate its safety, immunogenicity, and protective efficacy. **Methods:** A five-gene-deleted variant PRV strain, PRV-JM-DF, was used as the vector backbone. Candidate genomic insertion sites and endogenous late promoters were systematically screened, followed by construction of recombinant PRVs expressing PRRSV GP2, GP3, GP4, GP5, and M individually or as tandem antigen constructs. Recombinant viruses were characterized for antigen expression, replication, and genetic stability. Safety and immunogenicity were initially evaluated in female BALB/c mice, and the leading candidates were further assessed for safety, immunogenicity, and protective efficacy in 18 Landrace pigs of either sex following challenge with highly pathogenic PRRSV HuN4. **Results:** The TK locus supported efficient foreign-gene expression, and pUL39 exhibited the highest activity among the tested endogenous late promoters. The recombinant viruses retained replication competence and genetic stability. Among the candidates, rPRV-C-4T induced robust PRRSV- and PRV-specific neutralizing antibody responses and showed no obvious adverse effects during the experimental observation period in pigs. Following HP-PRRSV HuN4 challenge, all rPRV-C-4T-immunized pigs survived and exhibited reduced viremia, viral shedding and tissue viral loads, together with markedly alleviated pathological lesions. **Conclusions:** rPRV-C-4T provided protection against homologous HP-PRRSV HuN4 challenge while maintaining PRV-directed immune responses, supporting its potential as a PRV-vectored multivalent vaccine candidate.

## 1. Introduction

According to the United Nations Department of Economic and Social Affairs, the global human population is projected to reach 8.5 billion by 2030, 9.7 billion by 2050, and 10.4 billion by 2100 [1]. This rapid population growth, coupled with the pressures of diminishing arable land and global climate change, poses a formidable challenge to global food security. Livestock-derived animal protein, which provides comprehensive nutrition and high-quality dietary protein, is pivotal in addressing this challenge. Consequently, the rising demand for animal protein has elevated the status of pork as a primary protein source, making the swine industry a critical component of the agricultural economy worldwide. Recent data indicate that the global pig inventory has risen to approximately 780 million pigs, up from 750 million in 2021 [2], underscoring the sector’s expanding role.

Porcine reproductive and respiratory syndrome virus (PRRSV), a single-stranded positive-sense RNA virus belonging to the family Arteriviridae, is the etiological agent of PRRS. This disease, first identified in North America in 1987, is characterized by reproductive failure in pregnant sows and respiratory distress in pigs of all ages, posing a persistent threat to the global swine industry [3,4]. The PRRSV genome comprises nine open reading frames (ORFs). ORF1a and ORF1b encode polypropteins that are proteolytically cleaved into 12 non-structural proteins (NSP1-NSP12), which are essential for viral replication [5]. The remaining ORFs encode structural proteins: ORF2a, ORF2b, and ORF3–7, which yield GP2, E, GP3, GP4, GP5, M, and N, respectively [6,7]. The major structural proteins include GP5, matrix (M), and nucleocapsid (N) proteins. GP5 and M typically form a disulfide-linked heterodimer, although GP5 homodimers have also been reported [8]. The minor glycoproteins GP2, GP3, and GP4 form a heterotrimeric complex, whereas non-glycosylated proteins E (ORF2b) and ORF5a are integral components of the virion [9,10].

A particularly virulent lineage, the highly pathogenic PRRSV (HP-PRRSV), emerged in China in 2006. HP-PRRSV infection is characterized by high fever (41–42 °C), generalized cutaneous erythema, severe respiratory disease, and high morbidity (50–100%) and mortality (20–100%) rates across all age groups [11,12,13]. The initial outbreak resulted in the loss of over one million pigs in 2006 alone; the virus subsequently spread rapidly to Southeast Asia, with the first report in Vietnam in 2007, and reached India in 2013 [11,14,15,16]. The HP-PRRSV HuN4 strain, isolated during this epidemic, has been established as a prototype owing to its high pathogenicity [11,14]. To date, HP-PRRSV strains remain endemic to China, with new variants continuously emerging. There is still a lack of safe and broadly effective vaccines.

The use of herpesviruses as vaccine vectors was pioneered by the initial report in 1984 of herpes simplex virus type 1 (HSV-1) expressing exogenous genes [17]. Pseudorabies virus (PRV), an alphaherpes virus with swine as its natural host, has since been developed as a promising vector platform [18,19,20,21,22]. The PRV genome is a large double-stranded DNA molecule of approximately 143 kb, consisting of unique long (UL) and short (US) regions, each flanked by internal and terminal repeat sequences (IRS and TRS) [23,24,25]. Its substantial coding capacity allows for the insertion of large foreign DNA sequences. The application of PRV as a vector began in 1986, when Keeler et al. first demonstrated the expression of a gIII–β-galactosidase fusion protein [26]. Subsequent studies have exploited PRV as a vector to express heterologous antigens from major porcine pathogens, including African swine fever virus (ASFV) CD2v, porcine circovirus type 2 (PCV2) antigens, and classical swine fever virus (CSFV) E2 [27,28,29].

However, this promising landscape was disrupted in 2011 by the emergence of variant PRV strains in China, which caused severe pseudorabies (PR) outbreaks in pig farms despite vaccination with the conventional Bartha-K61 vaccine, leading to substantial economic losses [30,31,32,33,34,35,36]. These outbreaks indicate that vaccine constructs based on conventional attenuated strains may lack efficacy or safety against emerging variants, highlighting the urgent need to develop novel vector systems derived from currently prevalent strains. In response, our laboratory isolated a variant strain, PRV-JM, in 2022 and subsequently constructed a live-attenuated candidate, PRV-JM-ΔEI92K (deleted for gE, gI, US9, US2, and TK), which demonstrated excellent safety and protective efficacy in pigs [37]. Building on this foundation, the present study utilized PRV-JM-ΔEI92K (hereafter referred to as PRV-JM-DF, where JM denotes the PRV-JM strain and DF indicates a five-gene-deleted derivative lacking gE, gI, US9, US2, and TK) as the vector backbone. First, we systematically screened for the optimal foreign gene insertion site and the most effective endogenous late promoter. Using CRISPR/Cas9 technology, we constructed a panel of recombinant viruses expressing PRRSV antigens (GP2, GP3, GP4, GP5, and M) individually or as tandem constructs (C-3T, 39-3T, C-4T, and 39-4T). These candidates were rigorously evaluated for their genetic stability, safety, and immunogenicity in mouse and piglet models. Our results demonstrated that rPRV-C-4T confers protection against homologous HP-PRRSV HuN4 challenge. This work provides a PRV-based vaccine design framework and experimental evidence supporting further development of multivalent vaccines against PRRSV.

## 2. Materials and Methods

### 2.1. Ethics Statement

All animal experiments were performed according to the animal protocols approved by the Subcommittee on Research Animal Care at the HVRI and were carried out in strict accordance with the recommendations of the Guide for the Care and Use of Laboratory Animals of the Ministry of Science and Technology of the People’s Republic of China (approval numbers: 250320-02-GR, 250526-01-GR, and 250605-01-GR). The experimental protocols were prepared and approved before initiation of the animal studies but were not prospectively registered in a public protocol registry.

### 2.2. Viruses and Cells

PK-15, HEK293T, Vero-E6, and MARC-145 cell lines were maintained in our laboratory. PK-15 cells (porcine kidney epithelial cells), HEK293T cells (human embryonic kidney cells), Vero-E6 cells (African green monkey kidney epithelial cells), and MARC-145 cells (African green monkey kidney-derived cells permissive for PRRSV infection) were cultured in Dulbecco’s modified Eagle medium (DMEM; Gibco, Grand Island, NY, USA) supplemented with 10% fetal bovine serum (FBS) and incubated at 37 °C under 5% CO_2_. PRV-JM-DF, a five-gene-deleted derivative of the PRV-JM strain lacking gE, gI, TK, US2, and US9, was constructed and maintained in our laboratory. A commercially available modified-live virus (MLV) vaccine based on the attenuated PRRSV-2 HuN4 strain was used as the commercial comparator.

### 2.3. Primers and Plasmids

The plasmids pUC19, pX330, and pEGFP-N were maintained in the laboratory. Plasmids pCDNA3.1-GP2, pCDNA3.1-GP3, pCDNA3.1-GP4, pCDNA3.1-GP5, pCDNA3.1-M, pCDNA3.1-3T, and pCDNA3.1-4T were synthesized by Beijing Ruibo Xingke Biotechnology Co. Ltd. All primers used for amplification, identification, and sgRNA construction were synthesized by Beijing Ruibo Xingke Biotechnology Co. Ltd. (Beijing, China) (Tables S1–S3).

### 2.4. Extraction of Full-Length Viral Genome

The virus was inoculated into a monolayer of Vero-E6 cells and cultured at 37 °C. When the cytopathic effect (CPE) affected at least 80% of the cells, the cells were centrifuged at 250× *g* for 10 min. The supernatant was discarded, and the pellet was resuspended in 10 mL lysis clarification medium (LCM) buffer and kept on ice for 10 min. The suspension was shaken for 20 min and transferred to a new centrifuge tube on ice. A total of 3 mL of Freon (1,1,2-trifluorotrichloroethane) was added and the mixture was shaken vigorously for 5 min. After centrifugation at 3000× *g* for 10 min, the aqueous supernatant was transferred to a 50 mL centrifuge tube and further clarified by centrifugation at 10,000× *g* for 10 min at 4 °C. 2.5 mL of LCM containing 5% glycerol was placed in a sterile ultracentrifuge tube, underlaid with 2.5 mL of LCM containing 45% glycerol, and overlaid with clarified supernatant. The samples were ultracentrifuged at 10,062× *g* for 2 h at 4 °C. The supernatant was carefully removed, and the pellet was resuspended in 4.7 mL of Tris-NaCl-EDTA (TNE) buffer. After thorough dispersion, 250 μL of 10% sodium dodecyl sulfate (SDS) and 1 mg of proteinase K were added and the mixture was incubated at room temperature for 3 min. An equal volume of phenol was added, mixed gently for 5 min, and centrifuged at 4000× *g* for 10 min. The aqueous phase was transferred to a new sterile centrifuge tube, and the extraction was repeated twice. The supernatant was collected, and genomic DNA was precipitated by adding 2 volumes of ice-cold absolute ethanol, followed by incubation at −20 °C for 30 min. The DNA was pelleted by centrifugation at 8000× *g* for 15 min at 4 °C, washed once with 75% ethanol, air-dried, and then dissolved in an appropriate volume of sterilized deionized water. DNA concentration and purity were determined spectrophotometrically, and aliquots were stored at −80 °C.

### 2.5. Construction of Recombinant Virus

Recombinant viruses were constructed using genome co-transfection. Briefly, Vero cells were seeded in 6-well plates and transfected at approximately 80% confluence with 2 µg of PRV-JM-DF genomic DNA, 1 µg of plasmid pX330-sgRNA, and 1 µg of pUC19-based transfer vector using lipo3000 transfection reagent (Thermo Fisher Scientific, Waltham, MA, USA). At 3–4 days post-transfection, when CPE reached approximately 80%, the cells were subjected to three thaw cycles to release the progeny virus. The harvested virus was serially diluted (10^−2^, 10^−3^, and 10^−4^) and inoculated onto monolayers of PK-15 cells in 6-well plates. After adsorption for 2 h at 37 °C under 5% CO_2_, the inoculum was removed, and cells were overlaid with minimum essential medium (MEM) containing 1% low-melting-point agarose and 2% FBS. Following incubation for 36 h, individual plaques were selected and subjected to two additional rounds of plaque purification in PK-15 cells. Purified viral clones were amplified and successful recombination was confirmed by PCR.

### 2.6. Indirect Immunofluorescence Assay (IFA)

PK-15 cells grown in 12-well plates were infected with recombinant virus at a dose of 500 TCID_50_ per well. At 48 h post-infection, the supernatant was removed, and the cells were washed three times with phosphate-buffered saline (PBS) (5 min per wash). The cells were fixed with 300 μL of 4% paraformaldehyde per well for 15 min at room temperature, followed by three washes with PBS containing 0.05% Tween-20 (PBST) (5 min each). Permeabilization was performed using 300 μL of 0.3% Triton X-100 per well for 10 min, after which the cells were washed three times with PBST. Non-specific binding was blocked with 2% BSA in PBST for 30 min at room temperature. The cells were then incubated with mouse anti-FLAG antibody (diluted 1:1000) for 2 h at room temperature. Following three washes with PBST, cells were incubated with FITC-conjugated goat anti-mouse secondary antibody (diluted 1:1000) for 1 h at 37 °C. After three final washes with PBST, fluorescence was observed and images were captured using a fluorescent microscope (ZEISS, Oberkochen, Germany).

### 2.7. Western Blot Analysis of Protein Expression of Recombinant Viruses

PK-15 cells grown in 6-well plates were infected with the recombinant virus at a dose of 1000 TCID_50_. At 48 h post-infection, cells were harvested by scraping and lysed in 100 μL of cell lysis buffer supplemented with 2 μL of protease inhibitor. After incubation on ice for 20 min with gentle tapping of the tube every 5 min, the lysates were centrifuged at 2000× *g* for 5 min to remove cellular debris. The supernatant was mixed with 5× SDS loading buffer, boiled for 10 min, and cooled on ice. Protein samples (10 μg per lane) were separated on a 10% SDS-PAGE gel at 80 V for 20 min, followed by 120 V for 60 min. The proteins were then transferred onto PVDF membranes using a wet-transfer system (Invitrogen, Thermo Fisher Scientific, Waltham, MA, USA) at 200 mA for 80 min. Membranes were blocked with 5% skim milk in PBST for 2 h at 37 °C, washed three times with PBST (10 min each). Membranes were incubated with primary antibodies diluted 1:2000 in blocking buffer, followed by incubation with IRDye 800-conjugated secondary antibodies diluted 1:15,000. Following three washes (10 min each), the signals were detected using an Odyssey CLx imaging system (LI-COR, Lincoln, NE, USA).

### 2.8. Dual-Luciferase Reporter Assay

HEK293T cells were cotransfected with the indicated promoter-reporter plasmids. At 24 h post-transfection, cells were lysed and luciferase activities of IFN-β-, ISG54-, ISG56-, NF-қB-, ISRE-reporters and TK-Renilla reporter were measured with a Dual-luciferase Reporter Assay System (Promega, Madison, MI, USA) according to the manufacturer’s instructions. The data were normalized to the transfection efficiency by dividing the firefly luciferase activity by the Renilla luciferase activity. Each experiment was performed in triplicate and repeated in three independent experiments, and representative data are shown.

### 2.9. In Vitro Growth Kinetics and Plaque Morphology

To assess viral growth, PK-15 cells cultured in 6-well plates were infected with recombinant or parental viruses at an MOI of 0.1. After adsorption for 2 h at 37 °C, the supernatant was removed, cells were washed once with PBS, and DMEM with 2%FBS was added. Supernatants were collected at 6, 12, 24, 36, 48, and 60 h post-infection, and virus titers were determined using a 50% tissue culture infection dose (TCID_50_) assay. Growth curves are plotted as the mean ln TCID_50_/mL ± SD from three independent experiments.

For plaque assays, PK-15 monolayers in 6-well plates were infected with serial dilutions of the virus. After 2 h of adsorption, the inoculum was replaced with an overlay comprising MEM with 1% low-melting-point agarose and 2% FBS. Plates were incubated for 72 h at 37 °C under 5% CO_2_, fixed with 4% paraformaldehyde, and stained with 1% crystal violet for 12 h. Plaque morphology and size were observed under a light microscope (ZEISS, Oberkochen, Germany).

### 2.10. Animals, Housing, and Husbandry

Four-week-old female BALB/c mice were purchased from Beijing Vital River Laboratory Animal Technology Co., Ltd. (Beijing, China). Thirty-five-day-old Landrace pigs of either sex were obtained from Jiecheng Farm, Daoli District, Harbin, China. All pigs were confirmed to be negative for PRV and PRRSV before enrollment by PCR.

All animals were acclimatized for 5 days before the experiments. Animals were maintained at 20–24 °C and 40–70% relative humidity under a 12 h light/12 h dark cycle, with free access to feed and water. Mice were housed five per cage. Pigs were housed by experimental group, with up to five per pen. Before viral challenge, animals were maintained in conventional animal facilities; following challenge, they were transferred to negative-pressure barrier facilities. No additional environmental enrichment was provided during the study.

### 2.11. Experimental Design and Randomization

The individual animal was considered the experimental unit. Animals were allocated to predefined experimental groups without computer-generated randomization. Due to the exploratory nature of this vaccine evaluation study, no formal a priori power calculation was performed. Histopathological evaluations were performed by investigators blinded to treatment groups. Group sizes were selected based on previous studies using comparable PRV/PRRSV animal models, experimental feasibility, and the principle of using the minimum number of animals necessary to obtain interpretable efficacy and safety data. No single primary outcome was prospectively designated for sample-size determination; vaccine efficacy was evaluated using predefined clinical, serological, virological, survival, and pathological outcomes. No additional predefined humane endpoints were established beyond routine clinical monitoring during the scheduled observation period. Animals were monitored daily for general condition and clinical signs throughout the study.

### 2.12. Evaluation of Safety and Immunogenicity in Mice

Four-week-old female BALB/c mice were allocated to eight experimental groups (*n* = 5 per group). Groups 1–4 were immunized intramuscularly with 10^6^ TCID_50_ of rPRV-C-3T, rPRV-39-3T, rPRV-C-4T, or rPRV-39-4T, respectively. Groups 5 and 6 received mixed-regimen immunizations. Group 5 received a mixture of rPRV-GP2, rPRV-GP3, rPRV-GP4, and rPRV-GP5, whereas Group 6 received a mixture of rPRV-GP2, rPRV-GP3, rPRV-GP4, rPRV-GP5, and rPRV-M, with each recombinant virus administered at the same dose as the corresponding single recombinant PRV immunization group. Group 7 received 10^6^ TCID_50_ of PRV-JM-DF, and group 8 received 100 μL of DMEM.

Mice were monitored daily for clinical signs and survival for 28 days after immunization. Blood samples were collected on day 28 for detection of antigen-specific antibodies and on day 35 for serum neutralization assays against HP-PRRSV HuN4. On day 35, all mice were challenged intramuscularly with 10^4^ TCID_50_ of PRV-JM and monitored for an additional 14 days.

### 2.13. Evaluating Immunogenicity and Protective Efficacy in Pigs

Thirty-five-day-old PRV- and PRRSV-negative Landrace pigs of either sex were allocated to four groups: rPRV-C-4T (*n* = 5), rPRV-GP2/3/4/5/M (*n* = 5), commercial attenuated PRRSV vaccine (*n* = 3), and DMEM control (*n* = 5). Pigs in the recombinant-virus groups were immunized intramuscularly with 1 mL of the corresponding recombinant virus at a dose of 1 × 10^6^ TCID_50_ per pig. The commercial attenuated PRRSV vaccine was administered according to the manufacturer’s instructions, whereas pigs in the control group received 1 mL of DMEM intramuscularly.

During the 35-day immunization period, animals were monitored daily for general clinical condition. Rectal temperatures were recorded daily during the first 14 days, and body weights were measured weekly. Nasal and anal swabs were collected weekly to monitor recombinant PRV shedding. Blood samples were collected before immunization (day 0) and on days 21, 28, and 35 post-immunization for serological analyses.

At 35 days post-immunization, all pigs were challenged intranasally with 1 × 10^5^ TCID_50_ of HP-PRRSV HuN4. Following challenge, rectal temperature and clinical signs were recorded daily, body weight was measured weekly, and survival was monitored for 28 days. Blood, nasal swabs, and oropharyngeal swabs were collected on days 0, 3, 5, 7, 11, 14, 21, and 28 post-challenge. Animals that died spontaneously during the observation period were necropsied as soon as possible. At the end of the observation period, surviving animals were deeply anesthetized with pentobarbital, followed by exsanguination and necropsy. Lung, tonsil, brain, and lymph-node tissues were collected for virological, gross pathological, and histopathological analyses.

### 2.14. Quantitative Real-Time PCR for Viral Load Detection

Viral nucleic acids were extracted from blood, swab samples (nasal and oropharyngeal), or tissue homogenates (tonsils, lungs, lymph nodes) using a commercial magnetic-bead-based virus DNA/RNA extraction kit, according to the manufacturer’s protocol. For RNA samples, complementary DNA (cDNA) was synthesized using a reverse transcription kit (TIANGEN Biotech (Beijing) Co., Ltd., Beijing, China) in a 20 µL reaction containing 1 µg of total RNA, 4 µL of 5 × Mix Buffer, and 1 µL of RTase. The reaction was performed at 42 °C for 15 min followed by 85 °C for 5 min. Quantitative PCR was carried out in a 20 µL reaction containing 10 µL of 2 × *Taq* Pro Multiplex probe Mix, 2 µL of Primer-F/R, 1 µL of cDNA, 1 µL *Taq* Man probe, and 6 µL of H_2_O. The thermal cycling conditions were 50 °C for 5 min, 95 °C for 3 min, followed by 40 cycles of 95 °C for 15 s and 60 °C for 30 s. Fluorescence data were collected at the annealing/extension step of each cycle.

### 2.15. Serum Neutralization Tests

For PRV neutralization, serum samples were heat-inactivated at 56 °C for 30 min and serially diluted 2-fold in DMEM. Each dilution was mixed with an equal volume of PRV-JM containing 200 TCID_50_ and incubated at 37 °C for 1 h. The serum-virus mixture was then transferred onto Vero cell monolayers in 96-well plates and monolayers in 96-well plates for 2 h at 37 °C under 5% CO_2_. Subsequently, the inoculum was replaced with DMEM containing 2% FBS and cells were cultured for 48 h at 37 °C. The CPE was examined under a microscope, and the neutralization titer was calculated using the Reed–Muench method.

The same serum inactivation and dilution steps were followed for PRRSV neutralization. Diluted sera were mixed with HP-PRRSV HuN4 (200 TCID_50_) and incubated at 37 °C for 1 h. The mixture was added to MARC-145 cell monolayers in 96-well plates, allowed to adsorb for 2 h, and then overlaid with DMEM containing 2% FBS. Cells were incubated for 96 h, after which CPE was scored. Finally, the serum neutralization potency was calculated using the Reed-Muench method.

### 2.16. Histopathological Examination

Histopathological examinations were performed as previously described [38]. Tissue samples were fixed in 4% neutral-buffered formalin solution for 24 h, embedded in paraffin, and sectioned at a thickness of 3 μm. The sections were stained with hematoxylin and eosin (H&E) and examined under a light microscope. Histopathological evaluation was performed by investigators blinded to the treatment groups. Lesions were assessed based on inflammatory cell infiltration, tissue structural damage, hemorrhage, and degeneration. The overall pathological severity was determined by integrating the extent and distribution of these lesions.

### 2.17. Statistical Analysis

Statistical analyses were performed using GraphPad Prism (GraphPad Software, version 11.1.0, San Diego, CA, USA). Data distribution was assessed using the Shapiro–Wilk test. Comparisons among three or more groups at a single time point were performed using one-way ANOVA when data met parametric assumptions; otherwise, the Kruskal–Wallis test followed by Dunn’s multiple-comparisons test was used. Longitudinal measurements, including body weight, rectal temperature, and viral loads measured at multiple time points, were analyzed using two-way repeated-measures ANOVA with appropriate multiple-comparisons correction. Survival curves were analyzed using the Kaplan–Meier method and compared using the log-rank test. A two-sided *p* value < 0.05 was considered statistically significant.

## 3. Results

### 3.1. Screening of Optimal Insertion Site and Promoter for PRV-JM-DF Backbone

Selection of an appropriate genomic insertion site and potent promoter is critical for achieving stable and high-level expression of foreign antigens in viral vector vaccines. While loci such as gI/gE, TK, and gG have been reported as permissible for heterologous gene insertion in classical PRV strains [28,29,39], their efficiency may vary in different genetic backgrounds. Given that the Bartha-K61 vaccine strain harbors natural deletions in US2, gE, gI, and US9, which suggests inherent genomic instability, we excluded this site and instead focused on three alternative genomic regions, including the TK locus, the gG–gD intergenic region (rPRV-EGFP-gG-gD), and the UL11–UL10 intergenic region (rPRV-EGFP-UL11-10).

**Figure 1:**
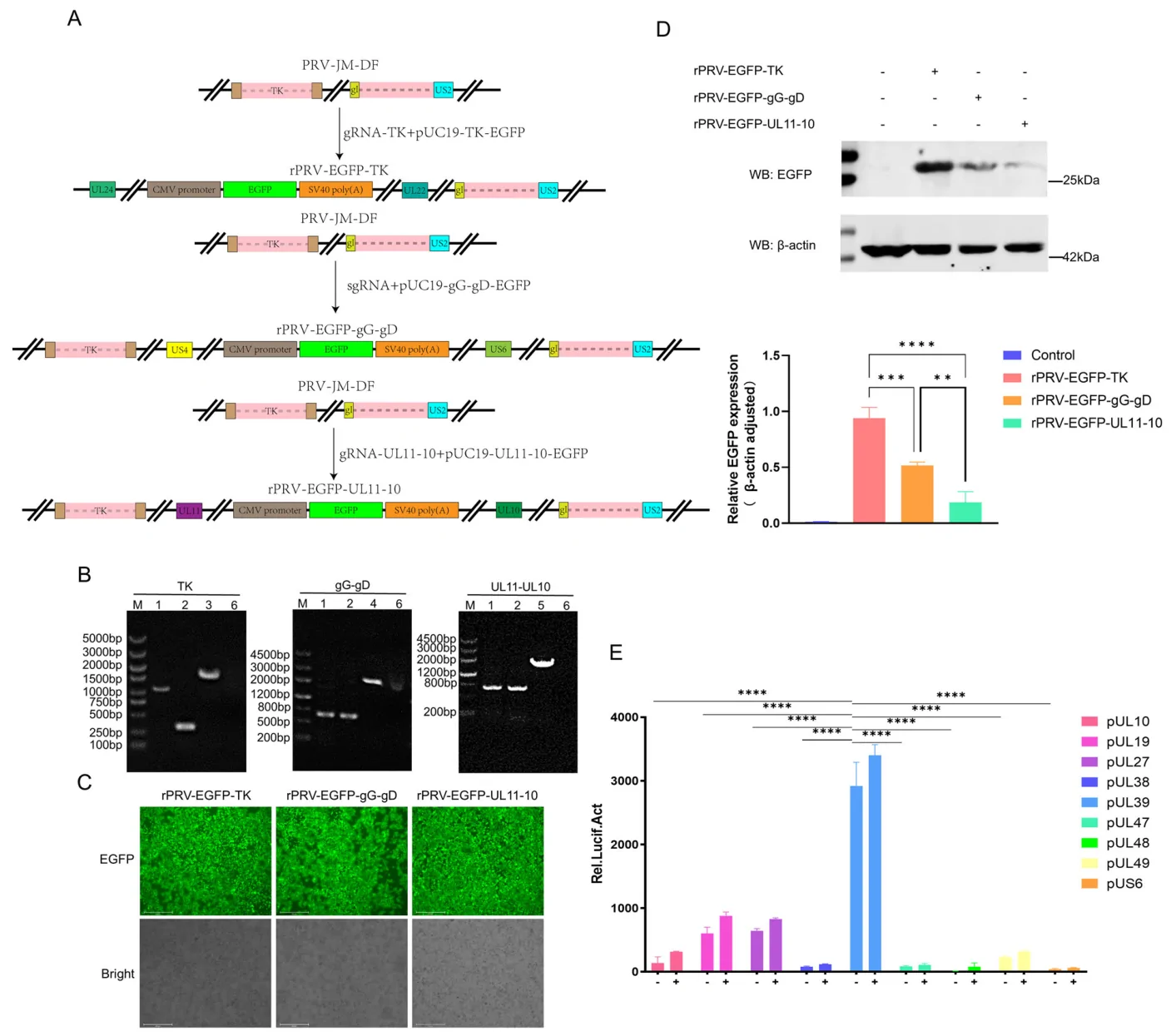
Screening of optimal insertion sites and promoters. (**A**) rPRV-EGFP-TK, rPRV-EGFP-gG–gD intergenic region (rPRV-EGFP-gG-gD), rPRV-EGFP-UL11-10 intergenic region (rPRV-EGFP-UL11-10), fluorescence reporter construction strategy. Arrows indicate the construction and recombination steps, and colored boxes represent the corresponding viral genomic elements and expression cassettes; (**B**) PCR identification of the constructed EGFP-expressing recombinants. Lanes: 1, PRV-JM genome; 2, PRV-JM-DF genome; 3, rPRV-EGFP-TK; 4, rPRV-EGFP-gG-gD; 5, rPRV-EGFP- UL11-10; 6, blank control. (**C**) Fluorescence microscopy of PK-15 cells infected with EGFP-expressing viruses. (**D**) Western blot analysis of EGFP expression in PK-15 cells infected with recombinant viruses. (**E**) Relative activities of nine candidate late PRV promoters measured using a dual-luciferase reporter assay. The standard deviations are shown as error bars. (** *p* < 0.01; *** *p* < 0.001; **** *p* < 0.0001). PRV, pseudorabies virus; PRV-JM-DF, five-gene-deleted PRV-JM strain lacking gE, gI, TK, US2, and US9; EGFP, enhanced green fluorescent protein; TK, thymidine kinase; gG, glycoprotein G; gD, glycoprotein D; UL, unique long region; CMV, cytomegalovirus promoter; SV40 poly(A), simian virus 40 polyadenylation signal.

To compare their suitability, we used CRISPR/Cas9-mediated homologous recombination to construct three recombinant viruses, each expressing EGFP at one of the candidate loci: rPRV-EGFP-TK, rPRV-EGFP-gG-gD, and rPRV-EGFP-UL11-10 (Figure 1A). The correct insertion was confirmed by PCR and sequencing (Figure 1B and Figure S1A). All three recombinants efficiently infected PK-15 cells and produced readily detectable fluorescence (Figure 1C). However, Western blot analysis revealed that EGFP expression was significantly higher when the gene was inserted at the TK locus than at the other two loci (Figure 1D). Therefore, the TK locus was established as the optimal insertion site for the PRV-JM-DF backbone vector platform.

Next, we aimed to identify a strong endogenous late promoter that drives antigen expression in a viral replication-dependent manner, potentially offering safer and more regulated expression than constitutive exogenous promoters (e.g., CMV/CAG). Based on genomic studies of PRV [40], we selected nine candidate late-gene promoters (pUL10, pUL19, pUL27, pUL38, pUL39, pUL47, pUL48, pUL49, and pUS6) for evaluation. A dual-luciferase reporter assay identified pUL39 as the most active promoter among those tested (Figure 1E). Further truncation analysis did not yield a fragment with higher activity than that of the full-length pUL39 sequence (Figure S1B,C). Consequently, the full-length pUL39 promoter was selected to drive foreign gene expression in the subsequent vaccine constructs.

### 3.2. Construction and In Vitro Characterization of Recombinant PRVs

**Figure 2:**
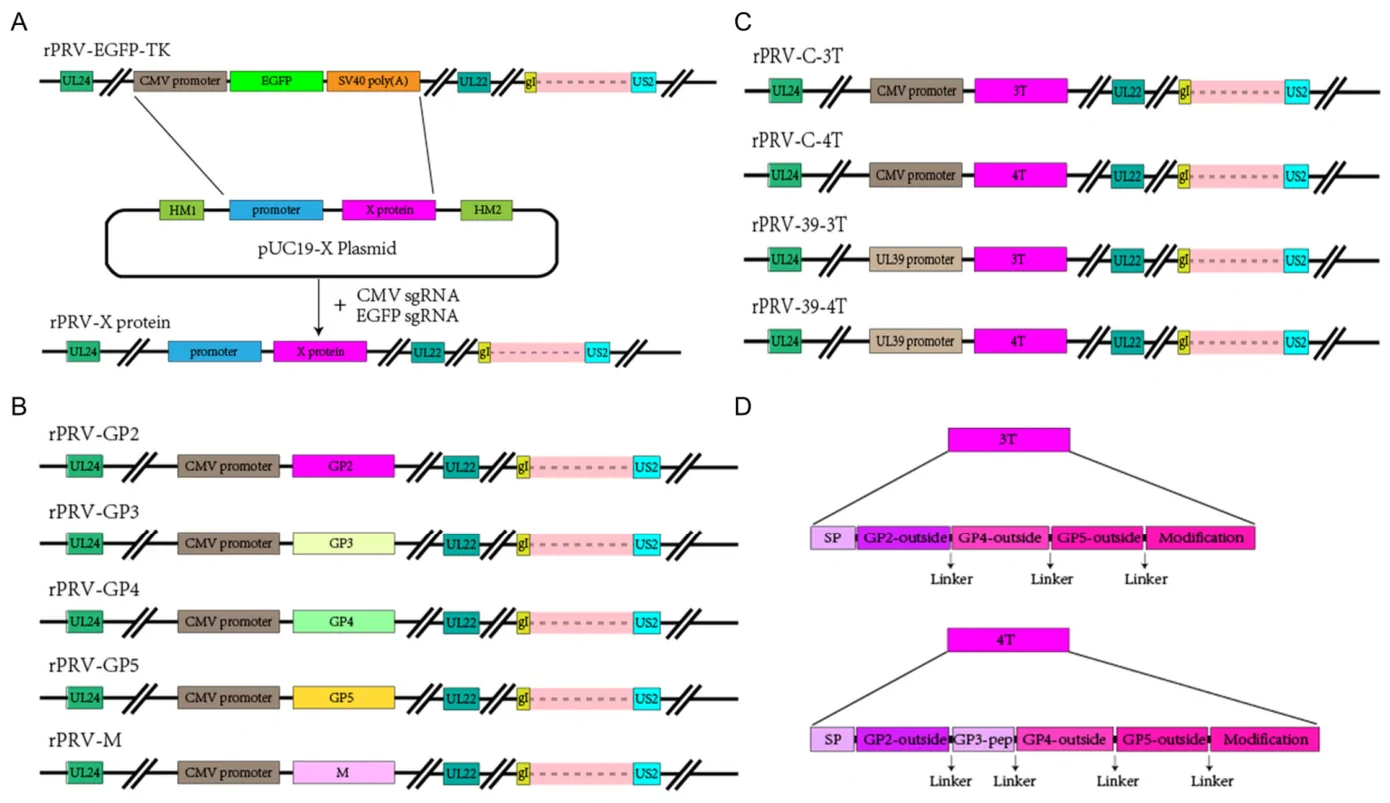
Construction strategy of recombinant virus expressing PRRSV antigens. (**A**) Schematic of recombinant virus construction based on the PRV-EGFP-TK backbone, using CRISPR/Cas9 mediated homologous recombination to replace the EGFP cassette with PRRSV antigen expression units at the TK locus. (**B**) Strategy for constructing recombinants expressing individual PRRSV structural proteins (GP2, GP3, GP4, GP5, or M). (**C**) Strategy for constructing recombinants (3T and 4T) driven by either the CMV (**C**) or pUL39 (39) promoter. (**D**) Modification strategy for the transmembrane domain of tandem PRRSV antigens. Different colors indicate different viral genes, promoters, expression proteins, and other genetic elements, as labeled in the schematics.

Based on the screening results, the optimal TK locus in the PRV-EGFP-TK backbone was selected for the insertion of PRRSV antigen expression cassettes (Figure 2A), utilizing the EGFP marker for efficient counter-selection. We pursued two distinct antigen presentation strategies: (i) a mixed-regimen strategy based on five single-gene recombinants (rPRV-GP2, -GP3, -GP4, -GP5, and-M) (Figure 2B), and (ii) a tandem antigen strategy. For the latter, the ectodomains of the PRRSV glycoproteins were fused at their N-termini to a signal peptide and at their C-termini to a transmembrane domain to facilitate proper glycosylation and membrane anchoring (Figure 2D). The expression of these antigens was controlled by either the exogenous CMV promoter or endogenous pUL39 promoter (Figure 2C). A C-terminal FLAG tag was incorporated into all the foreign gene constructs to enable immunological detection.

**Figure 3:**
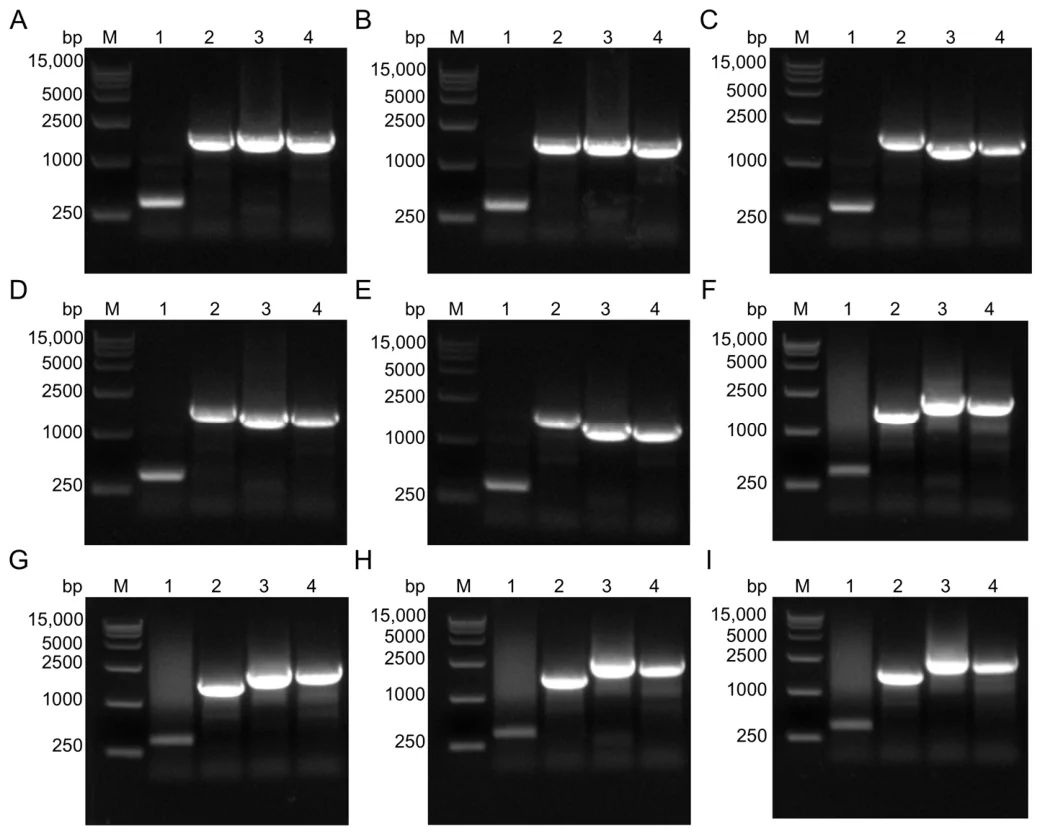
PCR identification of recombinant viruses. PCR verification of successful construction of the recombinant viruses using specific primers. (**A**) rPRV-GP2, (**B**) rPRV-GP3, (**C**) rPRV-GP4, (**D**) rPRV-GP5, (**E**) rPRV-M, (**F**) rPRV-C-3T, (**G**) rPRV-39-3T, (**H**) rPRV-C-4T, and (**I**) rPRV-39-4T. Lanes in each panel: 1, PRV-JM-DF genome; 2, rPRV-EGFP-TK genome; 3, positive plasmid control; and 4, genomic DNA of the corresponding recombinant virus.

**Figure 4:**
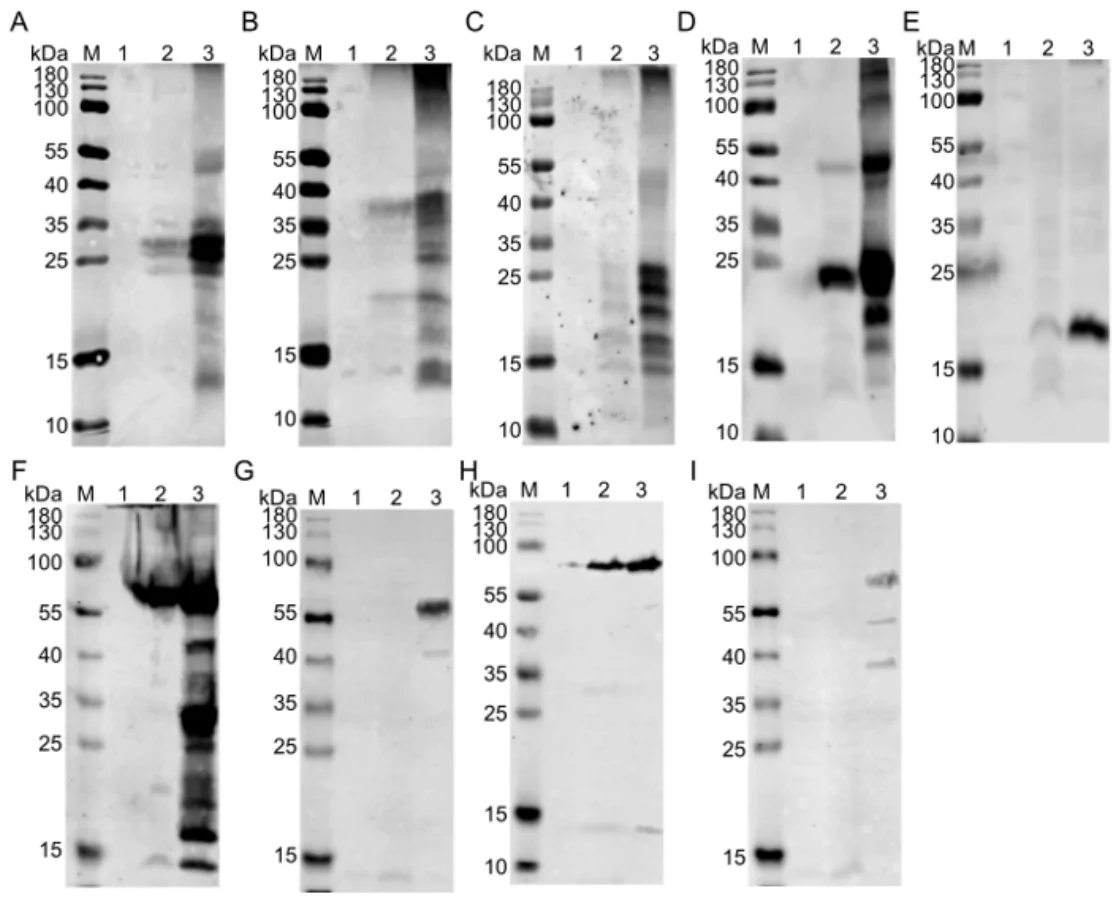
Western Blot analysis of recombinant virus protein expression. PK-15 cells were infected with the indicated recombinant viruses, and antigen expression was analyzed at 48 h post-infection. (**A**) rPRV-GP2; (**B**) rPRV-GP3; (**C**) rPRV-GP4; (**D**) rPRV-GP5; (**E**) rPRV-M; (**F**) rPRV-C-3T; (**G**) rPRV-39-3T; (**H**) rPRV-C-4T; and (**I**) rPRV-39-4T. For each panel, M indicates the molecular-weight marker; lane 1, mock-treated cell lysate; lane 2, lysate of cells transfected with the corresponding antigen-expression plasmid; and lane 3, lysate of cells infected with the corresponding recombinant PRV. The recombinant PRRSV proteins were detected using mouse anti-FLAG antibody (1:2000), followed by IRDye 800-conjugated secondary antibody (1:15,000).

The successful construction of all nine recombinant viruses (rPRV-GP2, -GP3, -GP4, -GP5, -M, -C-3T, -C-4T, -39-3T, and -39-4T) was confirmed by PCR and sequencing (Figure 3A–I). Protein expression in infected PK-15 cells was verified by indirect immunofluorescence assay (IFA) and Western blot, which detected the respective PRRSV proteins (Figure S2 and Figure 4A–I). Notably, expression of constructs driven by the pUL39 promoter was observed exclusively in virus-infected cells and not in cells transfected with the corresponding plasmid alone (Figure 4G,I), confirming its strict dependence on viral regulation as a viral late promoter.

### 3.3. Growth Characteristics and Genetic Stability of Recombinant PRVs

**Figure 5:**
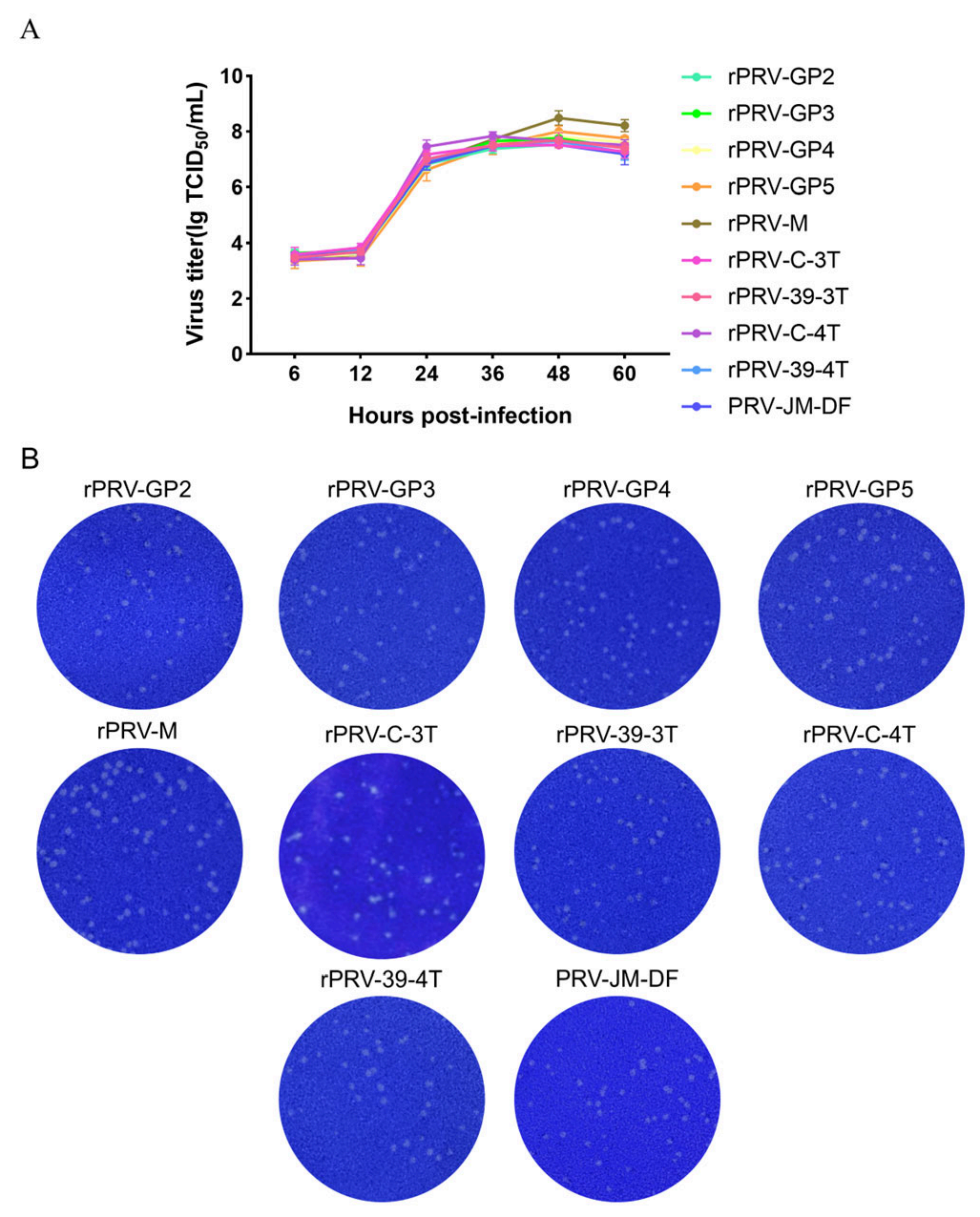
Growth characteristics of recombinant viruses. (**A**) One-step growth curve. PK-15 cells were infected with the indicated viruses at an MOI of 0.1. Cells were harvested at designated time points and viral titers were determined in PK-15 cells. The results represent three independent experiments. (**B**) Plaque morphology of PK-15 cell monolayers infected with the indicated viruses, overlaid with agarose, and stained with crystal violet at 72 h post-infection.

To assess whether the insertion of PRRSV antigen expression cassettes affected viral fitness, we analyzed the growth kinetics and plaque morphology of all recombinant viruses. One-step growth curve analysis revealed that, with the exception of rPRV-M, which showed a marginally higher titer, all recombinants replicated with kinetics comparable to that of the parental PRV-JM-DF virus (Figure 5A). Similarly, plaque assays demonstrated that the recombinants formed plaques with sizes and morphologies that were indistinguishable from those of the parental virus (Figure 5B), indicating that genetic modifications did not significantly impair viral replication or cell-to-cell spread.

To evaluate genetic stability, each recombinant virus was serially passaged into PK-15 cells for 30 generations. PCR analysis performed every fifth passage confirmed the stable maintenance of the inserted PRRSV antigen genes without detectable deletions or rearrangements (Figure S3). Collectively, these results demonstrate that the PRV-JM-DF backbone harboring foreign gene insertions at the TK locus retains excellent genetic stability and replication competence.

### 3.4. Safety and Immunogenicity of Recombinant PRVs in Mice

**Figure 6:**
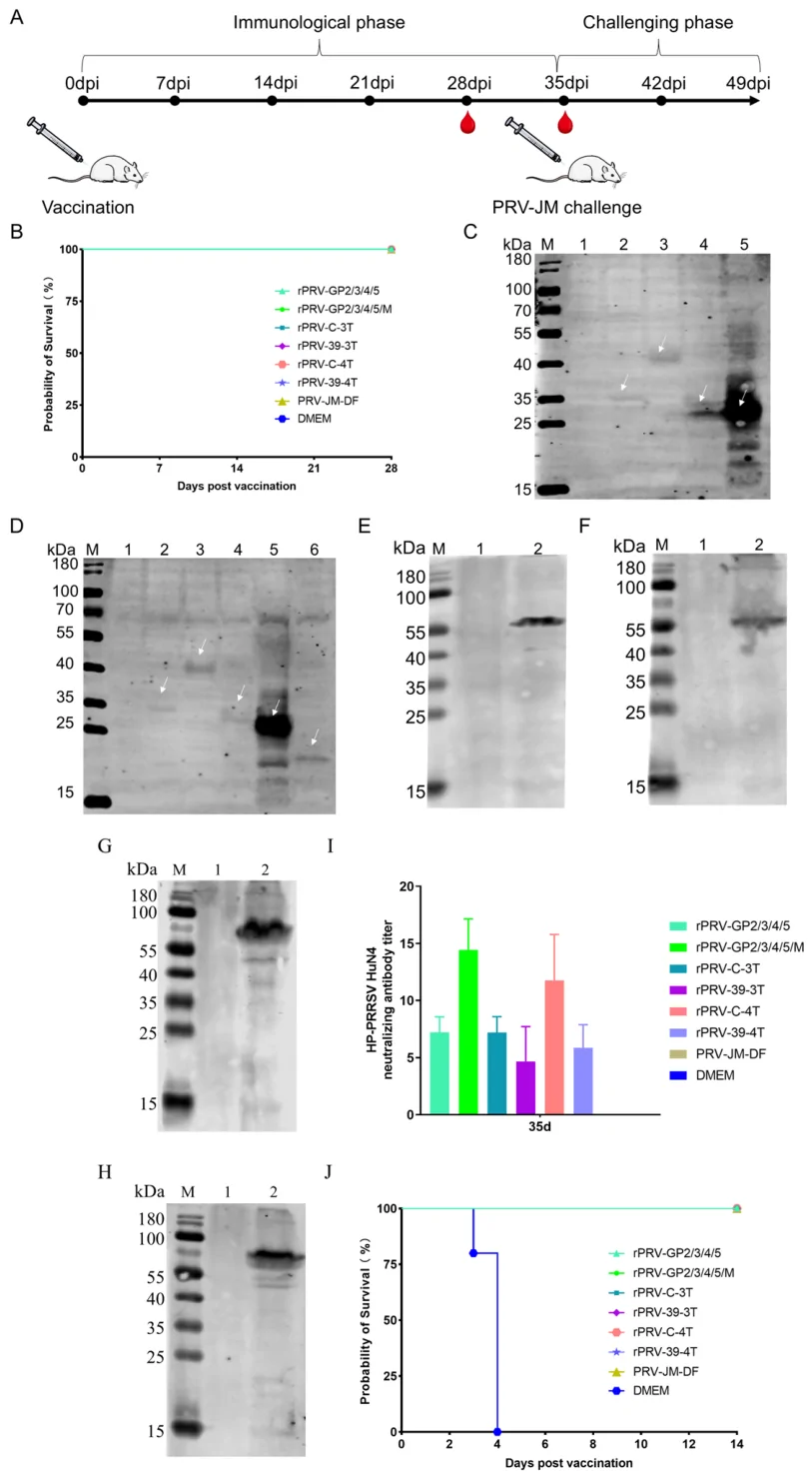
Safety and immunogenicity of recombinant viruses in mice. (**A**) Mice (*n* = 5 per group) were immunized with mixed regimens (rPRV-GP2/3/4/5 or rPRV-GP2/3/4/5/M), single recombinant viruses (rPRV-C-3T, rPRV-39-3T, rPRV-C-4T, rPRV-39-4T), the parental vector PRV-JM-DF, or DMEM. On day 35, mice were challenged and observed for 14 days. (**B**) Survival of mice during the 28-day immunization period. (**C**–**H**) Western blot analysis of antigen-specific antibodies in mouse sera collected on day 28. For each panel: Lane 1, mock-transfected cell lysate; Lanes 2–6, lysates from cells transfected with the indicated antigen-expression plasmids (pUC19-GP2, -GP3, -GP4, -GP5, -M, -C-3T, -39-3T, -C-4T, or -39-4T). Western Blot analysis of antigen-specific antibodies in mouse sera collected on day 28. For each panel: lanes 1, mock-transfected cell lysate; Lanes 2–6, lysates from cells transfected with the indicated antigen-expression plasmids (pUC19-GP2, -GP3, -GP4, -GP5, -M, -C-3T, -39-3T, -C-4T, and-39-4T). (**I**) Serum neutralization titers against HP-PRRSV HuN4 on day 35 post immunization. (**J**) Survival of mice following PRV-JM challenge (14 days post challenge).

To evaluate the safety and immunogenicity of the vaccine candidates, groups of mice (*n* = 5) were immunized with either mixed regimens (rPRV-GP2/3/4/5 or rPRV-GP2/3/4/5/M) or with single recombinant viruses expressing tandem antigens (rPRV-C-3T, -39-3T, -C-4T, and-39-4T). Control groups received either the parental vector PRV-JM-DF or DMEM (Figure 6A). All immunized mice remained healthy throughout the observation period, with no recorded clinical signs or mortality (Figure 6B), indicating that no obvious adverse effects were observed following administration of the recombinant constructs during the observation period.

Sera collected on day 28 post-immunization contained antibodies that specifically reacted with the respective PRRSV antigens in the Western blot analysis (Figure 6C–H), confirming successful antigen expression and humoral immunogenicity in vivo. Serum neutralization tests against HP-PRRSV HuN4 performed on day 35 showed that the mixed regimen rPRV-GP2/3/4/5/M and single recombinant rPRV-C-4T induced the highest neutralizing antibody titers (Figure 6I). Additionally, all vaccinated mice survived a subsequent challenge with the virulent PRV-JM strain without exhibiting clinical signs (Figure 6J), confirming that the recombinant strains retained the full protective efficacy of the parental vector against PRV.

### 3.5. Protective Efficacy of Recombinant PRVs in Pigs

**Figure 7:**
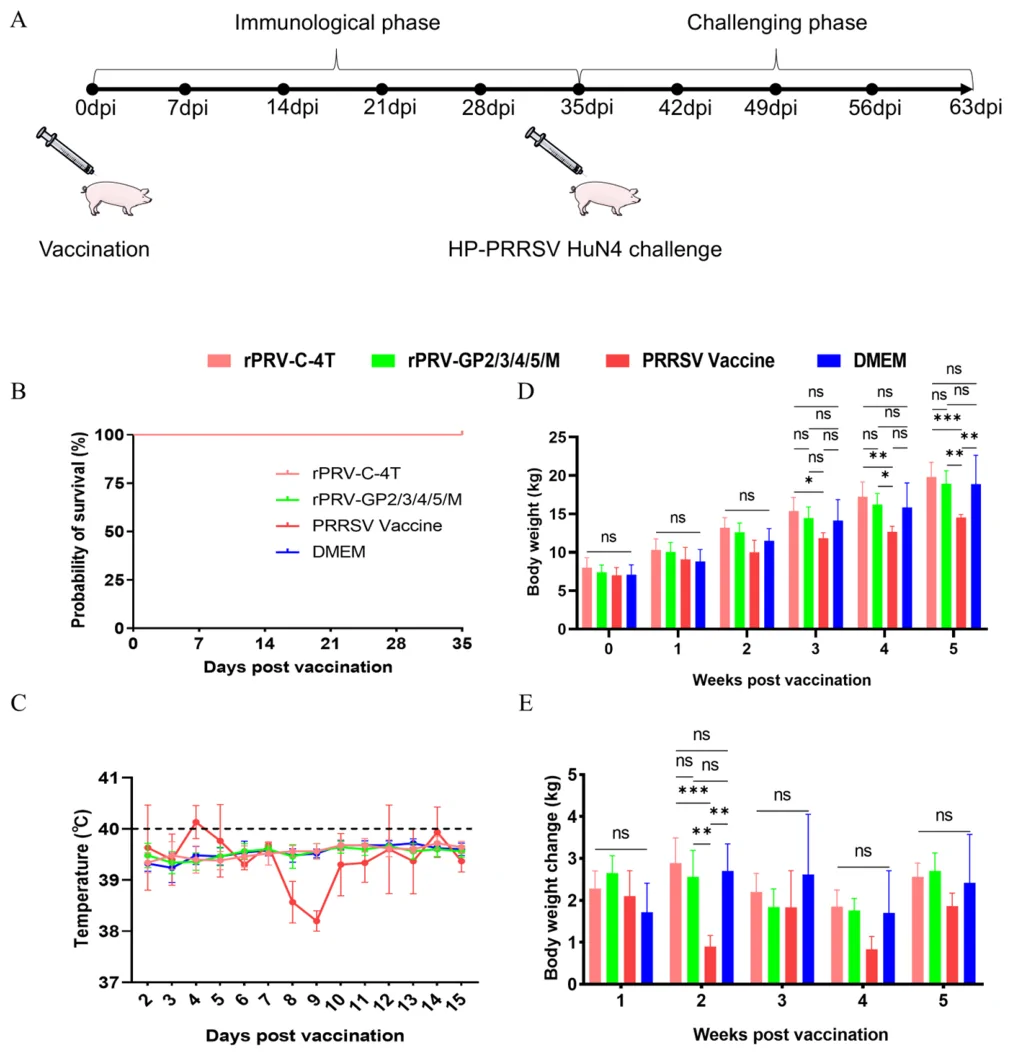

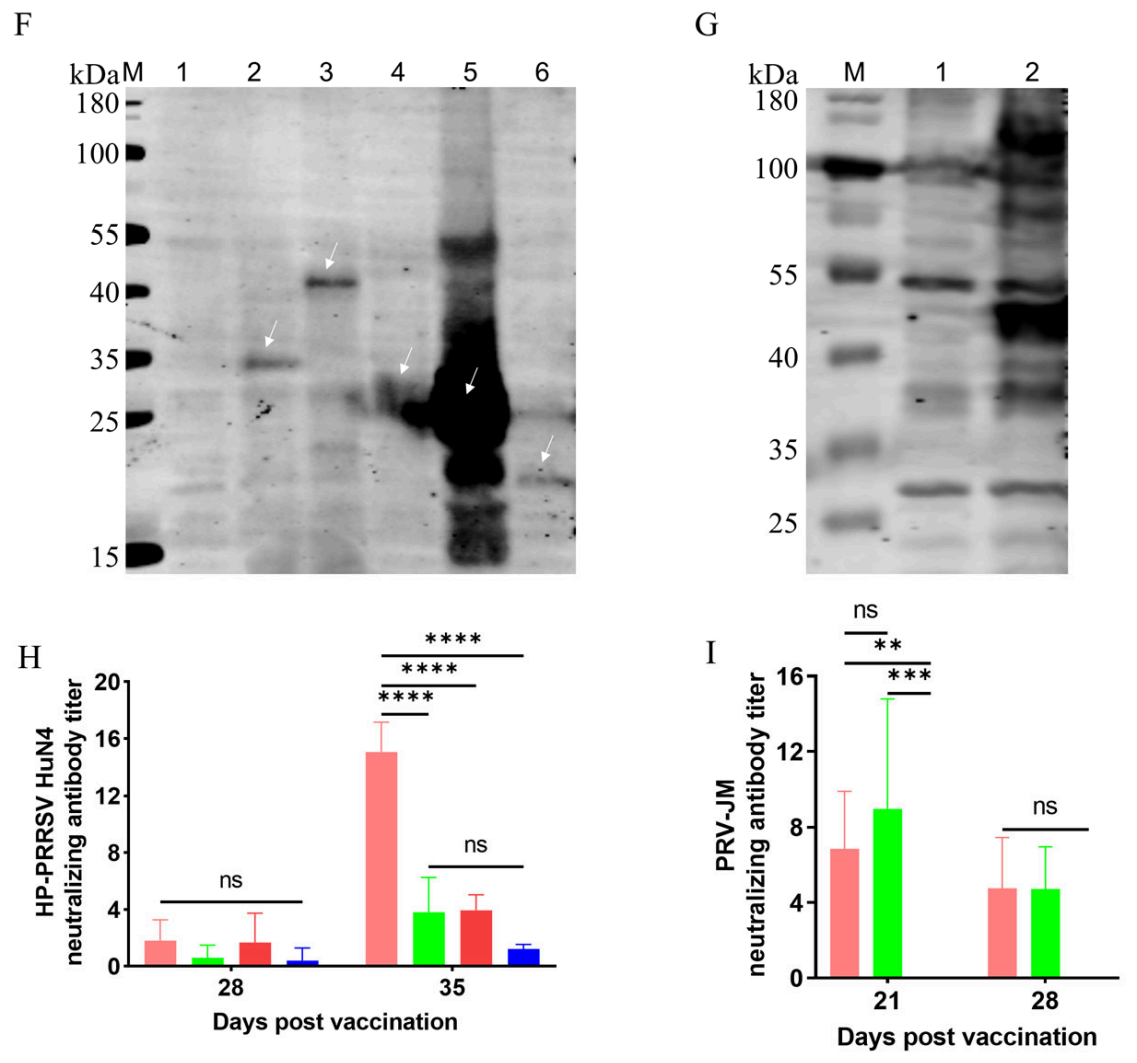
Immunogenicity and safety of recombinant virues in pigs. (**A**) Experimental timeline and group design. Pigs were immunized with rPRV-GP2/3/4/5/M (*n* = 5), rPRV-C-4T (*n* = 5), commercial attenuated PRRSV vaccine (*n* = 3), or DMEM (*n* = 5). All the groups were challenged with HP-PRRSV HuN4 on day 35. (**B**) Survival during the immunization phase. (**C**) Body weight and (**D**) rectal temperature recorded during the immunization period. (**E**) Weight gain rate during the immunization phase. (**F**,**G**) Western blot analysis of antigen-specific antibodies in post-immunization pig sera. (**F**) Sera from the rPRV-GP2/3/4/5/M group. (**G**) Sera from the rPRV-C-4T group. Lanes 1, mock-transfected cell lysate; 2–6 (**F**,**G**), lysates from cells transfected with the indicated antigen-expression plasmids (pUC19-GP2, -GP3, -GP4, -GP5, -M, or -C-4T). White arrows indicate the expected bands of the target proteins. (**H**) Serum neutralizing antibody titers against HP-PRRSV HuN4 on days 28 and 35. (**I**) Serum neutralizing antibody titers against PRV-JM were determined in pigs immunized with rPRV-GP2/3/4/5/M, rPRV-C-4T, and DMEM control on days 21 and 28 post-immunization. PRV-JM neutralization was not evaluated in the commercial PRRSV vaccine groups because these animals did not receive a PRV-based immunogen. (* *p* ≤ 0.05; ** *p* < 0.01; *** *p* < 0.001; **** *p* < 0.0001; ns, not significant).

Based on the promising results from the mouse study, two top-performing candidates, the mixed regimen rPRV-GP2/3/4/5/M and the single recombinant rPRV-C-4T, along with a commercial attenuated PRRSV vaccine and DMEM control, were selected for evaluation in pigs (Figure 7A). During the immunization period, pigs receiving the commercial PRRSV vaccine exhibited notable adverse reactions, including significant body temperature fluctuations, transient lameness, and diarrhea (Figure 7B–E). In contrast, pigs immunized with rPRV-JM-DF-based candidates remained clinically normal with stable body temperatures and unimpaired weight gain (Figure 7B–E). Furthermore, viral shedding of recombinant PRV in serum, nasal, and anal swabs was minimal or undetectable by four weeks post-immunization (Figure S4), indicating limited dissemination of the vaccine virus. Serological analyses confirmed the robust immunogenicity of the PRV-vectored candidates. PRRSV-specific antibodies were detected by Western blot in sera from both immunization groups (Figure 7F,G). Serum neutralization assays demonstrated PRRSV HuN4-neutralizing antibody responses in the immunized groups (Figure 7H). In addition, PRV-JM-neutralizing antibodies were detected in pigs receiving the two PRV-based recombinant vaccine regimens (Figure 7I). Together, these data underscore the favorable safety profile and potent immunogenicity of recombinant PRV vaccines in pigs.

**Figure 8:**
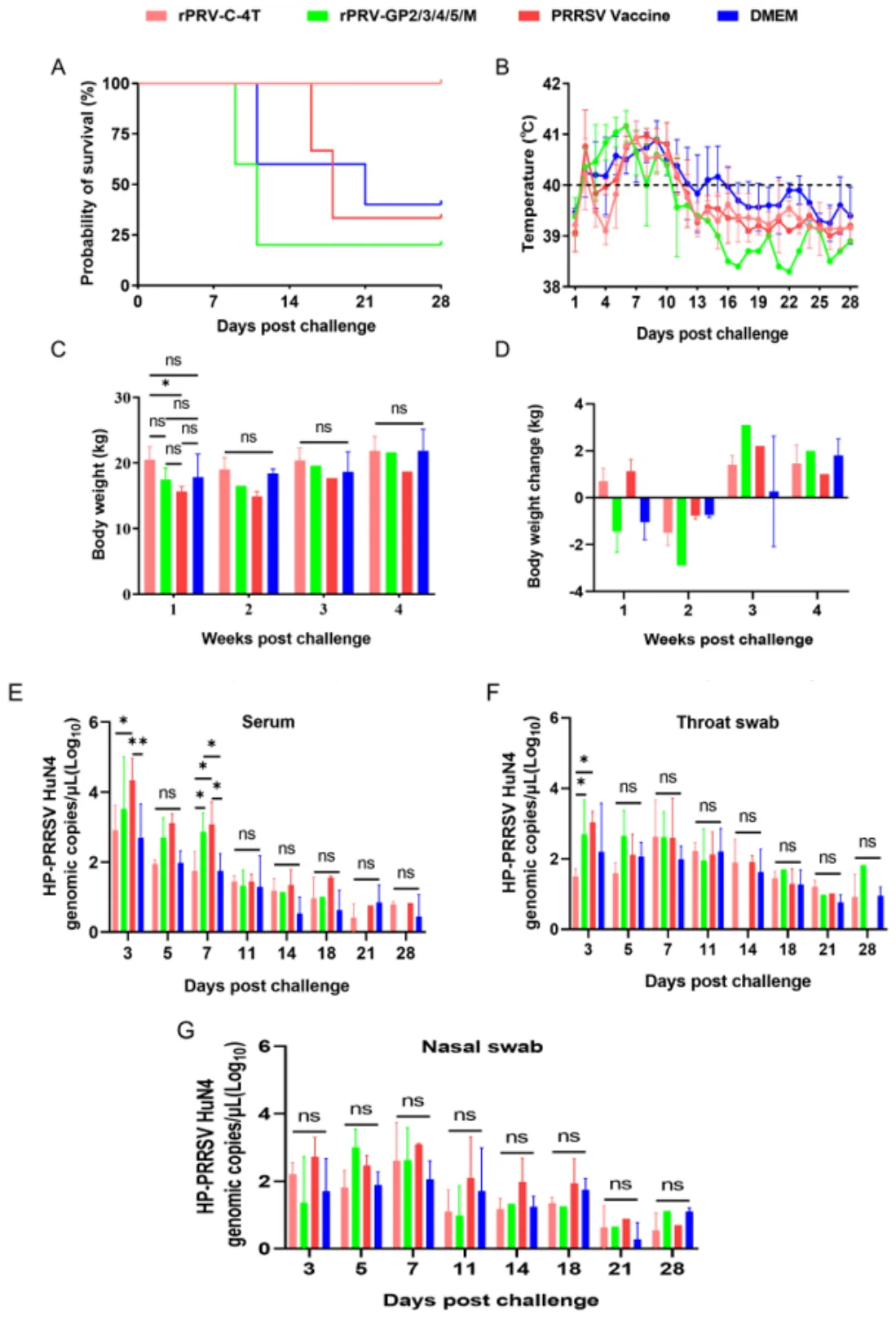

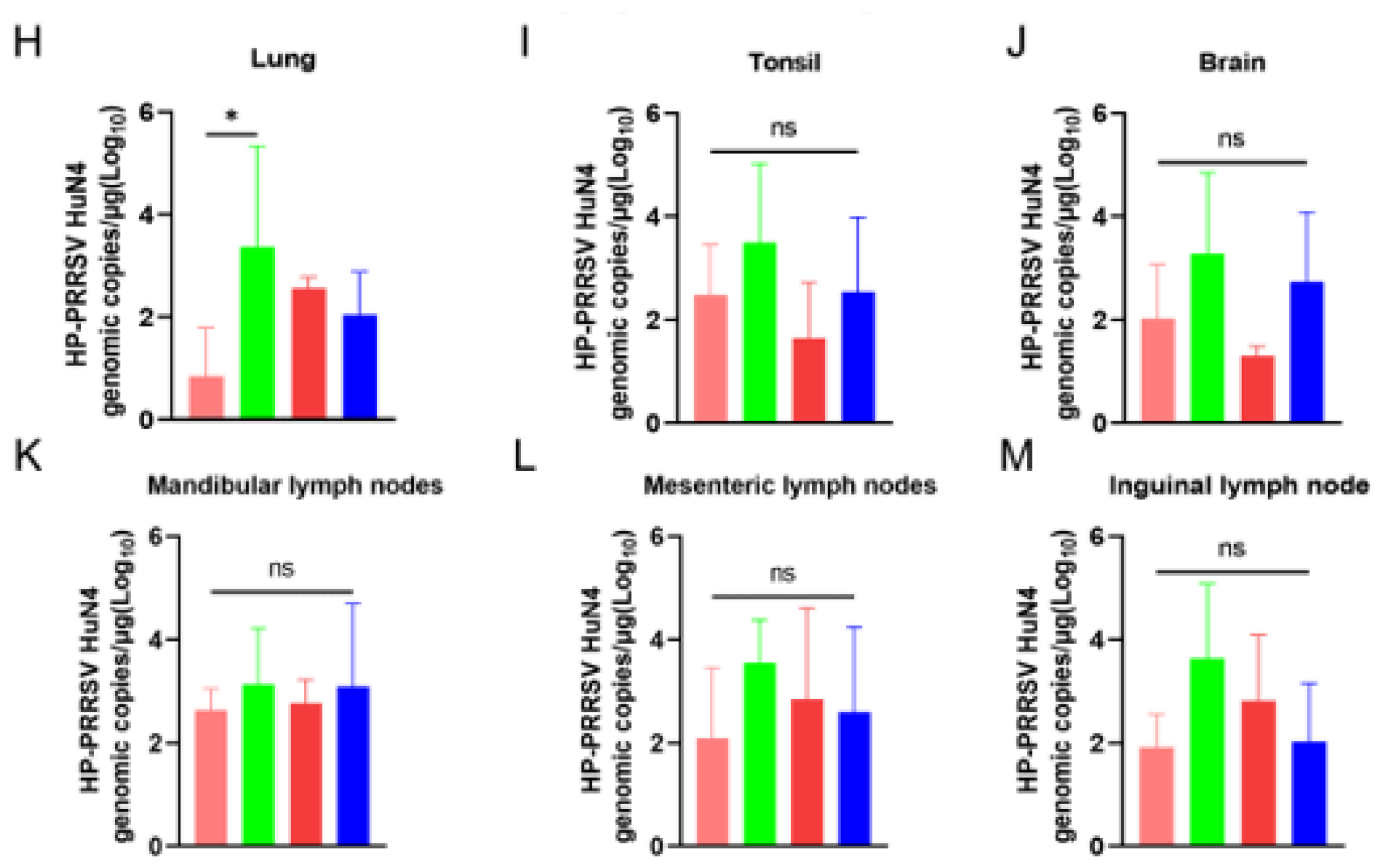
Protective efficacy of recombinant PRVs against HP-PRRSV challenge in pigs. (**A**) Survival over 28 days post-challenge; (**B**) Mean daily rectal temperature and (**C**) body weight during the challenge period; (**D**) Changes in body weight of pigs at 28 days post-challenge; (**E**–**G**) viral loads (**E**) in serum, (**F**) oropharyngeal swabs, and (**G**) nasal swabs at indicated time points; (**H**–**M**) Tissue viral loads in (**H**) lungs, (**I**) tonsils, (**J**) brain, (**K**) mandibular lymph nodes, (**L**) mesenteric lymph nodes, and (**M**) inguinal lymph nodes at necropsy. (ns, not significant; * *p* ≤ 0.05; ** *p* < 0.01).

Following the challenge with HP-PRRSV HuN4, survival rates, clinical signs, and virological parameters were systematically monitored (Figure 8A–D). During the acute phase (days 0–14 post challenge), piglets in the DMEM control and rPRV-GP2/3/4/5/M groups experienced high mortality, with 2/5 and 4/5 animals succumbing by days 12 and 11, respectively. This high mortality was consistent with the exceptionally high virulence of the HP-PRRSV HuN4 strain, which has been reported to cause 100% morbidity and 40–100% mortality in experimentally infected piglets [11,14]. The 40% mortality in the DMEM control group therefore fell within the expected range of this challenge model, whereas the even higher mortality (4/5) in the rPRV-GP2/3/4/5/M group indicated incomplete protection conferred by this recombinant. In contrast, all pigs in the rPRV-C-4T group as well as those in the commercial PRRSV vaccine group survived the initial 14-day period. Notably, the differential survival coincided with the neutralizing antibody responses measured at day 35 post-immunization: the rPRV-GP2/3/4/5/M regimen failed to elicit serum neutralizing antibodies above the DMEM background level (Figure 7H), whereas the rPRV-C-4T group developed markedly higher titers (approximately 1:16). Beyond day 14, sporadic late mortalities occurred in the DMEM control group (one pig on day 21) and commercial PRRSV vaccine group (two pigs on days 16 and 18). Post-mortem analysis confirmed that these late deaths were attributable to secondary *Haemophilus parasuis* infection rather than to direct PRRSV pathogenesis. PRRSV-induced immunosuppression is known to predispose pigs to secondary bacterial infections such as *Haemophilus parasuis* [41,42]. Notably, no secondary bacterial infections were observed in the rPRV-C-4T group, indicating preserved immune competence in these animals.

Among the surviving animals, the rPRV-C-4T group exhibited the most favorable clinical course, characterized by the most rapid resolution of fever and the lowest levels of viremia. The rPRV-C-4T group generally exhibited lower viral loads in serum, oropharyngeal swabs, and nasal swabs than the other groups, although statistically significant differences were restricted to selected time points, particularly during the early phase after challenge (Figure 8E–G). Consistent with these findings, post-mortem quantification of viral loads in tissues (lungs, tonsils, and lymph nodes) confirmed that the rPRV-C-4T group maintained the lowest viral burden, with a particularly marked reduction observed in the lung tissue (Figure 8H–M).

**Figure 9:**
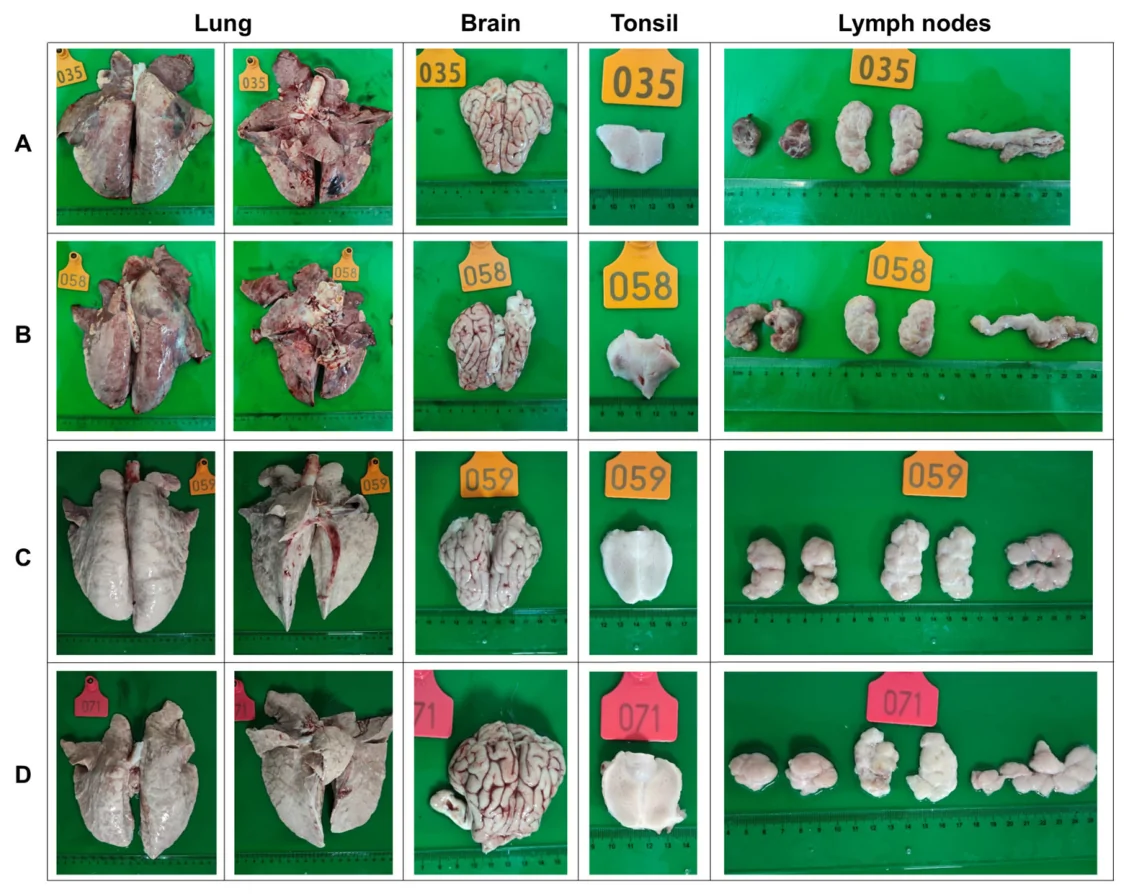
Gross pathology of major organs. Representative images of lung and lymph node lesions from (**A**) DMEM control, (**B**) rPRV-GP2/3/4/5/M, (**C**) rPRV-C-4T, and (**D**) commercial PRRSV vaccine groups at necropsy.

**Figure 10:**
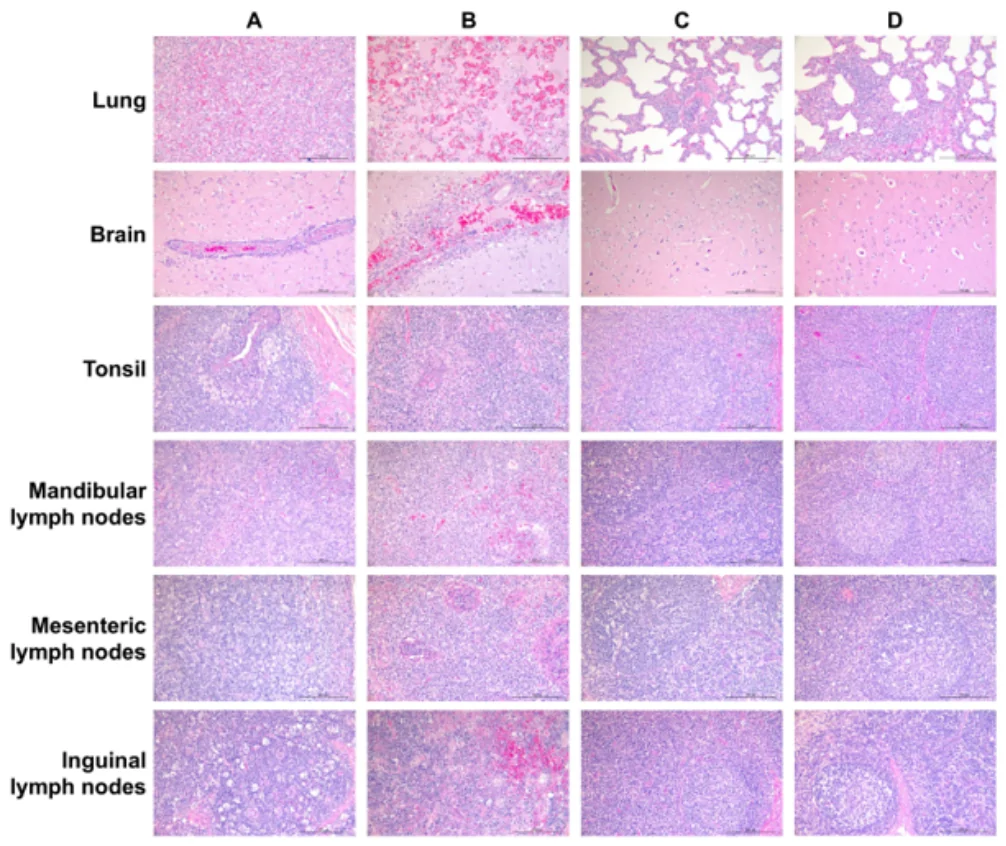
Histopathological examination (H&E staining). Representative micrographs of lung, tonsil, and lymph node sections from (**A**) DMEM control, (**B**) rPRV-GP2/3/4/5/M, (**C**) rPRV-C-4T, and (**D**) commercial PRRSV vaccine groups. Scale bars, 200 μm.

Gross pathological examination revealed severe pulmonary consolidation and lymph node hemorrhage in the DMEM control and rPRV-GP2/3/4/5/M groups, respectively. In contrast, neither the rPRV-C-4T nor the commercial PRRSV vaccine group exhibited obvious gross lesions (Figure 9). The histopathological analysis (H&E staining) corroborated these observations (Figure 10). The DMEM control and rPRV-GP2/3/4/5/M groups displayed severe and diffuse tissue damage across all the examined organs. In contrast, the rPRV-C-4T group showed only minimal pathological changes, limited to mild perivascular inflammation in the lungs and a slight reduction in tonsillar lymphocytes, with other organs remaining histologically normal. The commercial vaccine group presented mild lesions.

In summary, these results demonstrated that rPRV-C-4T is safe, highly immunogenic, and confers effective protection against homologous HP-PRRSV challenge, thereby establishing it as a promising live viral vector vaccine candidate.

## 4. Discussion

In this study, we developed a rationally engineered PRV-based vector platform for PRRSV antigen delivery. By systematically comparing insertion sites, promoter configurations, and antigen architectures, we identified the TK locus as a favorable site for stable foreign antigen expression, and established tandem PRRSV glycoprotein delivery as a favorable strategy compared with a mixed monovalent-vector regimen. The lead candidate, rPRV-C-4T, retained parental PRV vector fitness and genetic stability, induced neutralizing antibodies against both PRV and PRRSV, while protecting pigs from lethal HP-PRRSV HuN4 challenge. These findings provided a functional design framework for PRV-vectored vaccines against antigenically complex swine viruses.

Advances in molecular biotechnology, particularly the CRISPR/Cas9-mediated genome-editing system, have profoundly accelerated vaccine research and development. CRISPR/Cas9 has emerged as a powerful tool for precise construction of PRV mutants. Previous studies have employed this system to generate PRV recombinants with targeted deletions in genes such as gE, gI, and TK [43,44,45]. Furthermore, integration of CRISPR/Cas9 with homologous recombination (HR) and bacterial artificial chromosome (BAC) techniques has significantly enhanced the efficiency of viral genome engineering [46,47]. Previous studies have constructed and evaluated two live-attenuated PRV candidated using CRISPR/Cas9 [37]. Based on its excellent safety and immunogenicity profile, we selected PRV-JM-DF as the vector backbone for exogenous gene insertion in the present work.

Because the optimal insertion site and promoter for achieving high-level exogenous protein expression can be strain-dependent, we first systematically screened for the most suitable genomic locus within the PRV-JM-DF backbone. While several studies have reported permissible insertion sites in PRV, such as those near gI/gE, TK, or gG, the reported efficiencies are inconsistent, likely reflecting genetic differences between PRV strains [28,29,39]. Our comparative analysis identified the TK locus as the optimal insertion site for the PRV-JM-DF vector, supporting its use in stable and high-yield antigen expression. Although strong exogenous promoters such as CMV are frequently employed for promoter selection, systematic evaluation of endogenous PRV promoters has been limited. In this study, we focused specifically on late viral promoters to couple antigen expression with viral replication, a strategy that may enhance safety by limiting antigen production in actively infected cells. Our screening identified pUL39 as the most active late promoter among the tested candidates. Although the expression levels driven by pUL39 were lower than those achieved with the CMV promoter and correlated with reduced antibody titers in mice, its strict replication-dependent regulation presents a distinct advantage for safety-oriented vector design. Thus, pUL39 represents a valuable alternative for applications in which regulated rather than maximal expression is desired, meriting further investigation in vaccine contexts where safety is paramount.

The immunodominance of PRRSV remains unclear. Early reports indicated that monoclonal antibodies targeting a putative “major neutralizing epitope” on GP5 often showed limited neutralizing activity [48]. Moreover, antibodies targeting the GP5-M heterodimer, which can bind to virions, frequently fail to mediate neutralization [49]. Peptide-scanning studies using the European prototype Lelystad virus have suggested that GP3, rather than GP5 or M, may be a primary target of neutralizing antibodies in convalescent sera [50]. Additional studies have identified GP4 as a key neutralization target in both PRRSV genotypes, potentially driving viral evolution [51,52,53]. Furthermore, GP2 harbors neutralizing epitopes [50]. These findings motivated our first vaccination strategy to construct five monovalent recombinant viruses, each expressing a single PRRSV protein (GP2, GP3, GP4, GP5, or M). We hypothesized that mixed immunization with these viruses would elicit a broader and potentially synergistic immune response, covering multiple neutralization targets. This approach also considers the biological context; GP2, GP3, and GP4 form a functional heterotrimeric complex that interacts with CD163 primarily through GP2 and GP4 [54]. Given that GP5 is the most abundant glycoprotein on the virion surface, it was prioritized in our design. In the second strategy, we engineered tandem antigen constructs (designated 3T and 4T), in which the ectodomains of these glycoproteins were concatenated and anchored to the membrane via transmembrane domains. This design aimed to present multiple antigenic domains in a single polypeptide, potentially facilitating a coordinated immune recognition and response. These tandem constructs were subsequently placed under the control of different promoters (CMV and pUL39) for comparative evaluation.

All the recombinant viruses successfully expressed the intended PRRSV antigens in infected PK-15 cells, with expression levels exceeding those achieved by plasmid transfection alone. Importantly, the insertion of these expression cassettes did not significantly impair viral replication, as evidenced by growth kinetics and plaque morphology comparable to those of the parental PRV-JM-DF. Serial passage experiments confirmed the genetic stability of all constructs over 30 generations, thereby validating PRV-JM-DF as a stable and robust viral vector and underscoring the suitability of the TK locus for high-level foreign gene expression.

In mouse immunization studies, all recombinant viruses demonstrated an excellent safety profile, even at an elevated total dose administered to the mixed-regimen groups. Each construct induced readily detectable antigen-specific antibodies and all vaccinated mice were fully protected against a subsequent lethal challenge with virulent PRV, confirming the retention of the vector’s intrinsic immunogenicity. Notably, serum neutralization assays against HP-PRRSV HuN4 revealed that the mixed regimen of rPRV-GP2/3/4/5/M and the single tandem-antigen virus rPRV-C-4T elicited the highest neutralizing antibody titers. Constructs utilizing the pUL39 promoter induced lower neutralizing titers than their CMV-driven counterparts, which is consistent with their lower antigen expression levels in vitro. The superior performance of rPRV-C-4T over the mixed-monovalent regimen suggests that antigen architecture and vector burden are critical determinants of PRV-vectored vaccine efficacy. Although the mixed regimen theoretically broadens antigenic coverage, the simultaneous administration of five replicating PRV vectors may introduce competition among vectors, unbalanced antigen expression, and excessive innate or inflammatory stimulation. In contrast, the tandem cassette ensures the co-delivery of multiple glycoprotein ectodomains within a single infected cell, potentially promoting more coordinated antigen presentation while minimizing the total vector dose. Based on these combined safety and immunogenicity data, rPRV-C-4T and rPRV-GP2/3/4/5/M mixed regimens were selected for further evaluation in pigs.

For the swine trial, we employed PRRSV- and PRV-free pigs to closely mimic field susceptibility, while controlling for pre-existing immunity. During immunization phase, pigs receiving the commercial attenuated PRRSV vaccine showed fever, lameness, and diarrhea during the observation period. In contrast, pigs immunized with either rPRV-JM-DF-based candidate showed no clinical abnormalities, maintained normal weight gain, and displayed safety profiles indistinguishable from the DMEM control group, underscoring the favorable tolerability of our vectored vaccines.

To rigorously assess protection, all pigs were challenged with a high dose (10^5^ TCID_50_) of HP-PRRSV HuN4. The rPRV-GP2/3/4/5/M group experienced the most prolonged febrile response and highest mortality among the vaccinated groups. The differential neutralizing antibody responses between the two vaccine strategies—background levels for the mixed regimen versus approximately 1:16 for rPRV-C-4T—are consistent with neutralizing antibodies serving as a correlate of protective immunity against PRRSV [55,56] and likely contributed to the differential survival observed after challenge. This outcome may reflect the complexity of administering multiple replication-competent recombinant PRV vectors simultaneously. Compared with the single recombinant vaccine regimen, the mixed formulation resulted in a higher cumulative vector exposure, which may have influenced host responses through increased antigenic burden, interactions among different recombinant viruses, or altered immune regulation. However, the present study was not specifically designed to determine the mechanism underlying the reduced survival observed in this group, and these possibilities remain speculative. Further studies involving controlled dose-comparison experiments and larger animal cohorts will be required to clarify the factors contributing to this observation. Pigs in the commercial PRRSV vaccine group also suffered from extended fever, and two pigs died during the later phase of the experiment. Post-mortem findings were consistent with secondary *Haemophilus parasuis* infection (Figure S5B), which was likely a consequence of challenge-induced immunosuppression.The absence of secondary bacterial infections in the rPRV-C-4T group further suggests that vaccination preserved normal immune competence, contrasting with the challenge-induced immunosuppression observed in the other groups. However, the comparison with the commercial PRRSV vaccine group should be interpreted cautiously because this comparator group contained only three animals, which limited the statistical power for direct comparison between vaccine candidates.

Notably, pigs in the rPRV-C-4T group exhibited a markedly attenuated clinical course, characterized by only mild and transient fever, no mortality, and unimpaired weight gain throughout the challenge period. Both gross pathology and histopathological examination revealed minimal lung changes, comparable in severity to those observed in the commercial vaccine group. Most importantly, viral load quantification demonstrated that the rPRV-C-4T group maintained significantly lower viral burdens in the serum and lung tissues, exhibiting reductions of up to 100-fold in some compartments compared to other groups. The higher neutralizing responses and superior protection achieved by the M-lacking rPRV-C-4T also suggest that the M protein did not contribute positively to protective efficacy in this vaccine design, although the precise contribution of M warrants further investigation given the additional differences between the two strategies. Collectively, these data demonstrate that rPRV-C-4T provides protection against homologous HP-PRRSV HuN4 challenge and supports its potential as a live viral vector vaccine candidate.

Although the HP-PRRSV HuN4 lineage used in the present challenge is no longer the predominant circulating strain in China, the vector platform and antigen-design strategy established here are potentially adaptable to contemporary PRRSV strains. Several limitations of the present study should be acknowledged. First, although histopathological examination provided supportive evidence for the protective effect of rPRV-C-4T, the current pathological evaluation was mainly qualitative, and future studies incorporating standardized quantitative lesion scoring systems will further strengthen pathological assessment. Second, the relatively small animal numbers, particularly in the commercial vaccine comparator group, together with the absence of prospective sample-size calculation and computer-generated randomization, may limit the statistical robustness of direct comparisons. Third, although robust antibody responses and neutralizing activity were demonstrated, cellular immune responses and other correlates of protection were not comprehensively evaluated. Fourth, protective efficacy was assessed using only a homologous HP-PRRSV HuN4 challenge model, and protection against contemporary heterologous PRRSV strains remains to be determined. Finally, although no obvious adverse effects were observed during the experimental period, long-term safety, viral shedding, environmental persistence, and field-level biosafety require further investigation. Therefore, further studies involving larger animal cohorts, contemporary heterologous PRRSV strains, and field conditions will be required to determine the breadth, reproducibility, and generalizability of protection provided by rPRV-C-4T.

## 5. Conclusions

In this study, we established a PRV-JM-DF-based vector platform for PRRSV antigen delivery by screening suitable genomic insertion sites and endogenous promoters and constructing recombinant PRVs expressing individual or tandem PRRSV antigens. The recombinant viruses showed stable antigen expression, replication competence, and genetic stability. Mouse experiments confirmed their safety and immunogenicity, and rPRV-C-4T and the rPRV-GP2/3/4/5/M mixed regimen showed the strongest neutralizing responses and were further evaluated in pigs. Notably, rPRV-C-4T showed the most favorable protective profile among the candidates evaluated in this study, providing complete survival after HP-PRRSV HuN4 challenge together with reduced clinical signs, viremia, viral shedding, tissue viral loads, and pathological lesions. These findings identify rPRV-C-4T as the leading vaccine candidate in this study and support its further development as a PRV-vectored multivalent vaccine.

## Data Availability

All data supporting the findings of this study are included in the published article and its Supporting Information Files. Additional datasets generated and analyzed during the current study are available from the corresponding author upon reasonable request.

## Supplementary Materials

The following supporting information can be downloaded at: https://www.mdpi.com/article/10.3390/vaccines14100915/s1; Figure S1: Identification of fluorescent virus and promoter truncation; Figure S2: Identification of the expression of exogenous proteins of recombinant virus by IFA; Figure S3: Identification of the genetic stability of recombinant viruses by PCR; Figure S4: Virus load of PRV in piglet immune period; Figure S5: Tissue damage in piglets post-challenge; Table S1: Primers used for constructing plasmids in this study; Table S2: Primers used for sgRNA in this study; Table S3: Primers used for qPCR in this study.
