# Supplementary material for: Construction of Recombinant PRV Expressing PRRSV Antigens and Evaluation of Their Protective Efficacy in Pigs Against Homologous Challenge
Source: Vaccines (Basel). 2026 Oct 21;14(10):915. doi: 10.3390/vaccines14100915 (PMC13709669; doi:10.3390/vaccines14100915)
Supplement: Supplementary file 1 [file vaccines-14-00915-s001.zip › Author Checklist - Full.pdf]

NOTE: Please save this file locally before filling in the table, DO NOT work on the file within your internet browser as changes will not be saved. Adobe Acrobat Reader (available free here) is recommended for completion.

The ARRIVE guidelines 2.0: author checklist

The ARRIVE Essential 10

These items are the basic minimum to include in a manuscript. Without this information, readers and reviewers cannot assess the reliability of the findings.

Item		Recommendation	Section/line number, or reason for not reporting
**Study design**	1	For each experiment, provide brief details of study design including:	
		a. The groups being compared, including control groups. If no control group has been used, the rationale should be stated.	Materials and Methods: Evaluation of Safety and Immunogenicity in Mice; Evaluation of Immunogenicity and Protective Efficacy in Pigs. All experimental and control groups are specified.
		b. The experimental unit (e.g. a single animal, litter, or cage of animals).	Materials and Methods: Experimental design and randomization. The individual animal was considered the experimental unit.
**Sample size**	2	a. Specify the exact number of experimental units allocated to each group, and the total number in each experiment. Also indicate the total number of animals used.	Materials and Methods: Evaluation of Safety and Immunogenicity in Mice; Evaluation of Immunogenicity and Protective Efficacy in Pigs. Mice: n = 5 per group, 8 groups (40 mice total). Pigs: n = 5, 5, 3, and 5 in the four groups (18 pigs total).
		b. Explain how the sample size was decided. Provide details of any *a priori* sample size calculation, if done.	Materials and Methods: Experimental design and randomization. No formal a priori power calculation was performed. Group sizes were based on previous comparable PRV/PRRSV studies, experimental feasibility, and the principle of using the minimum number of [illegible]
**Inclusion and exclusion criteria**	3	a. Describe any criteria used for including and excluding animals (or experimental units) during the experiment, and data points during the analysis. Specify if these criteria were established *a priori*. If no criteria were set, state this explicitly.	Materials and Methods: Experimental design and randomization. No predefined inclusion or exclusion criteria were applied after group allocation.
		b. For each experimental group, report any animals, experimental units or data points not included in the analysis and explain why. If there were no exclusions, state so.	Materials and Methods: Experimental design and randomization. No animals or available data points were excluded; data unavailable after spontaneous animal death could not be collected at subsequent time points, and data [illegible]
		c. For each analysis, report the exact value of *n* in each experimental group.	Materials and Methods: animal experiment sections; Results and Figures 6–8.
**Randomisation**	4	a. State whether randomisation was used to allocate experimental units to control and treatment groups. If done, provide the method used to generate the randomisation sequence.	Materials and Methods: Experimental design and randomization. Animals were allocated to experimental groups without a computer-generated randomization sequence.
		b. Describe the strategy used to minimise potential confounders such as the order of treatments and measurements, or animal/cage location. If confounders were not controlled, state this explicitly.	Materials and Methods: Experimental design and randomization. No specific allocation strategy was used to control potential confounders such as cage/pen location or the order of treatment and measurement.
**Blinding**	5	Describe who was aware of the group allocation at the different stages of the experiment (during the allocation, the conduct of the experiment, the outcome assessment, and the data analysis).	Materials and Methods: Experimental design and randomization. Investigators were aware of group allocation during animal allocation, immunization, viral challenge, clinical monitoring, outcome assessment, and data analysis; no blinding was performed.
**Outcome measures**	6	a. Clearly define all outcome measures assessed (e.g. cell death, molecular markers, or behavioural changes).	Materials and Methods: animal experiment sections; Quantitative Real-Time PCR for Viral Load Detection; Serum Neutralization Tests; Histopathological Examination. Outcomes included survival, clinical signs, rectal temperature, body weight [illegible]
		b. For hypothesis-testing studies, specify the primary outcome measure, i.e. the outcome measure that was used to determine the sample size.	Materials and Methods: Experimental design and randomization. No single primary outcome was prospectively designated for sample-size determination; vaccine efficacy was assessed using predefined clinical, serological, virological, survival, and [illegible]
**Statistical methods**	7	a. Provide details of the statistical methods used for each analysis, including software used.	Materials and Methods: Statistical Analysis.
		b. Describe any methods used to assess whether the data met the assumptions of the statistical approach, and what was done if the assumptions were not met.	Materials and Methods: Statistical Analysis. Data distribution was assessed using the Shapiro–Wilk test; parametric or non-parametric analyses were selected according to distributional assumptions.
**Experimental animals**	8	a. Provide species-appropriate details of the animals used, including species, strain and substrain, sex, age or developmental stage, and, if relevant, weight.	Materials and Methods: Animals, housing, and husbandry. Four-week-old female BALB/c mice and 35-day-old Landrace pigs of either sex were used.
		b. Provide further relevant information on the provenance of animals, health/immune status, genetic modification status, genotype, and any previous procedures.	Materials and Methods: Animals, housing, and husbandry. Mice were obtained from Beijing Vital River Laboratory Animal Technology Co., Ltd.; pigs were obtained from Jiecheng Farm, Daoli District, Harbin. Pigs were confirmed negative for PRV and [illegible]
**Experimental procedures**	9	For each experimental group, including controls, describe the procedures in enough detail to allow others to replicate them, including:	
		a. What was done, how it was done and what was used.	Materials and Methods: Evaluation of Safety and Immunogenicity in Mice; Evaluation of Immunogenicity and Protective Efficacy in Pigs; Quantitative Real-Time PCR; Serum Neutralization Tests; Histopathological [illegible]
		b. When and how often.	Materials and Methods: mouse and pig experiment sections. Timing and frequency of immunization, challenge, clinical monitoring, body-weight measurement, temperature measurement, blood collection, and swab [illegible]
		c. Where (including detail of any acclimatisation periods).	Materials and Methods: Animals, housing, and husbandry. Animals were acclimatized for 5 days; housing conditions and transfer to negative-pressure barrier facilities after challenge are described [illegible]
		d. Why (provide rationale for procedures).	Introduction, final paragraph; Materials and Methods: animal experiment sections. Mouse studies were used for initial safety and immunogenicity evaluation, followed by protective-efficacy assessment of selected [illegible]
**Results**	10	For each experiment conducted, including independent replications, report:	
		a. Summary/descriptive statistics for each experimental group, with a measure of variability where applicable (e.g. mean and SD, or median and range).	Results and Figures 6–10. Group-level outcomes and variability are presented where applicable.
		b. If applicable, the effect size with a confidence interval.	Effect sizes with confidence intervals were not reported.

The Recommended Set

These items complement the Essential 10 and add important context to the study. Reporting the items in both sets represents best practice.

Item		Recommendation	Section/line number, or reason for not reporting
**Abstract**	11	Provide an accurate summary of the research objectives, animal species, strain and sex, key methods, principal findings, and study conclusions.	Abstract. The objectives, animal models, key m
**Background**	12	a. Include sufficient scientific background to understand the rationale and context for the study, and explain the experimental approach.<br>b. Explain how the animal species and model used address the scientific objectives and, where appropriate, the relevance to human biology.	Introduction.<br>Introduction, particularly the final paragraph. The rationale for PRV as a vaccine vector and the use of mouse and pig models for vaccine evaluation are described.
**Objectives**	13	Clearly describe the research question, research objectives and, where appropriate, specific hypotheses being tested.	Abstract: Background/Objectives; Introduction,
**Ethical statement**	14	Provide the name of the ethical review committee or equivalent that has approved the use of animals in this study, and any relevant licence or protocol numbers (if applicable). If ethical approval was not sought or granted, provide a justification.	Materials and Methods: Ethics statement. Animal protocols were approved by the Subcommittee on Research Animal Care at the HVRI (approval numbers 250320-02-GR, 250526-01-GR, and 250605-01-GR).
**Housing and husbandry**	15	Provide details of housing and husbandry conditions, including any environmental enrichment.	Materials and Methods: Animals, housing, and
**Animal care and monitoring**	16	a. Describe any interventions or steps taken in the experimental protocols to reduce pain, suffering and distress.<br>b. Report any expected or unexpected adverse events.<br>c. Describe the humane endpoints established for the study, the signs that were monitored and the frequency of monitoring. If the study did not have humane endpoints, state this.	Materials and Methods: Evaluation of Immunogenicity and Protective Efficacy in Pigs. Surviving pigs were deeply anesthetized with pentobarbital before exsanguination and necropsy.<br>Results: Protective efficacy of recombinant PRVs in pigs. Observed adverse events included fever, lameness, diarrhea, mortality, and secondary Haemophilus parasuis infection; no clinical signs or mortality were observed in immunized mice.<br>Materials and Methods: animal experiment sections. No additional predefined humane endpoints were established beyond routine clinical monitoring; animals were monitored daily for clinical signs and general condition.
**Interpretation/ scientific implications**	17	a. Interpret the results, taking into account the study objectives and hypotheses, current theory and other relevant studies in the literature.<br>b. Comment on the study limitations including potential sources of bias, limitations of the animal model, and imprecision associated with the results.	Discussion.<br>Discussion, final paragraph. Limitations include the homologous HP-PRRSV HuN4 challenge, relatively small group sizes, and lack of blinding.
**Generalisability/ translation**	18	Comment on whether, and how, the findings of this study are likely to generalise to other species or experimental conditions, including any relevance to human biology (where appropriate).	Discussion, final paragraph. The need for validation using larger cohorts, contemporary heterologous PRRSV strains, and field conditions is discussed.
**Protocol registration**	19	Provide a statement indicating whether a protocol (including the research question, key design features, and analysis plan) was prepared before the study, and if and where this protocol was registered.	Materials and Methods: Ethics statement. Experimental protocols were prepared and approved before the animal studies but were not prospectively registered in a public protocol registry.
**Data access**	20	Provide a statement describing if and where study data are available.	Data availability statement.
**Declaration of interests**	21	a. Declare any potential conflicts of interest, including financial and non-financial. If none exist, this should be stated.<br>b. List all funding sources (including grant identifier) and the role of the funder(s) in the design, analysis and reporting of the study.	Conflicts of Interest. The authors declare no conflicts of interest.<br>Funding. All funding sources and grant identifiers are reported. The funders had no role in study design, data collection, data analysis, interpretation of the results, manuscript preparation, or the decision to publish.
