# Supplementary material for: Construction of Recombinant PRV Expressing PRRSV Antigens and Evaluation of Their Protective Efficacy in Pigs Against Homologous Challenge
Source: Vaccines (Basel). 2026 Oct 21;14(10):915. doi: 10.3390/vaccines14100915 (PMC13709669; doi:10.3390/vaccines14100915)
Supplement: Supplementary file 1 [file vaccines-14-00915-s001.zip › vaccines-4538747-Supplementary Figures.pdf]

Supplementary Data

Construction of Recombinant PRV Expressing PRRSV Antigens and Evaluation of Their Protective Efficacy in Pigs Against Homologous Challenge

Shanghui Wang [1,†], Yunfei Liu [1,†], Longfei Han [1,†], Liguo Gao [1], Guangqiang Ye [1], Xiang Zhao [1], Yi Liu [1], Hongyang Liu [1,2], Zhaoxia Zhang [1,2,*], Changjiang Weng [1,2,*]

1 Division of Fundamental Immunology, National African Swine Fever Para-Reference Laboratory, State Key Laboratory for Animal Disease Control and Prevention, Harbin Veterinary Research Institute, Chinese Academy of Agricultural Sciences, Harbin 150069, China; wangsh19961128@163.com (S.W.); l910776357@foxmail.com (Y.L.); hanlongfei453@163.com (L.H.); gaoliguo369@outlook.com (L.G.); ygqyyds123@163.com (G.Y.); 17808318103@163.com (X.Z.); liuyi_microbio@163.com (Y.L.); liuhongyang@caas.cn (H.L.)

2 Heilongjiang Provincial Key Laboratory of Veterinary Immunology, Harbin 150069, China

* Correspondence: wengchangjiang@caas.cn (C.W.); zhangzhaoxia@caas.cn (Z.Z.)

† These authors contributed equally to this work.

Figure S1

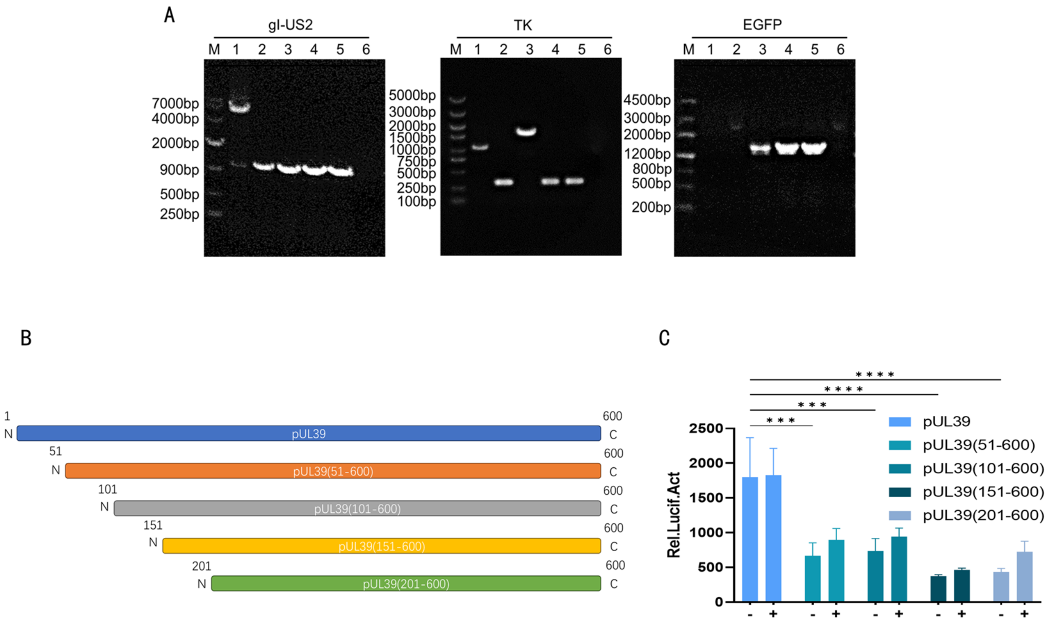

Figure S1. Identification of fluorescent virus and promoter truncation.

(A) PCR detection of recombinant fluorescent virus gI-US2 deletion, TK deletion, and EGFP fragment. Line 1: PRV-JM genome; Line 2: PRV-JM-DF genome; Line 3: PRV-JM-DF-EGFP-TK genome; Line 4: PRV-JM-DF-EGFP-gG-gD genome; Line 5: PRV-JM-DF-EGFP-gG-gD genome; Line 6: blank control. (B) Schematic diagram of pUL39 promoter truncation. (C) Activity detection of pUL39 promoter truncation (*** $p < 0.001$; **** $p < 0.0001$).

Figure S2

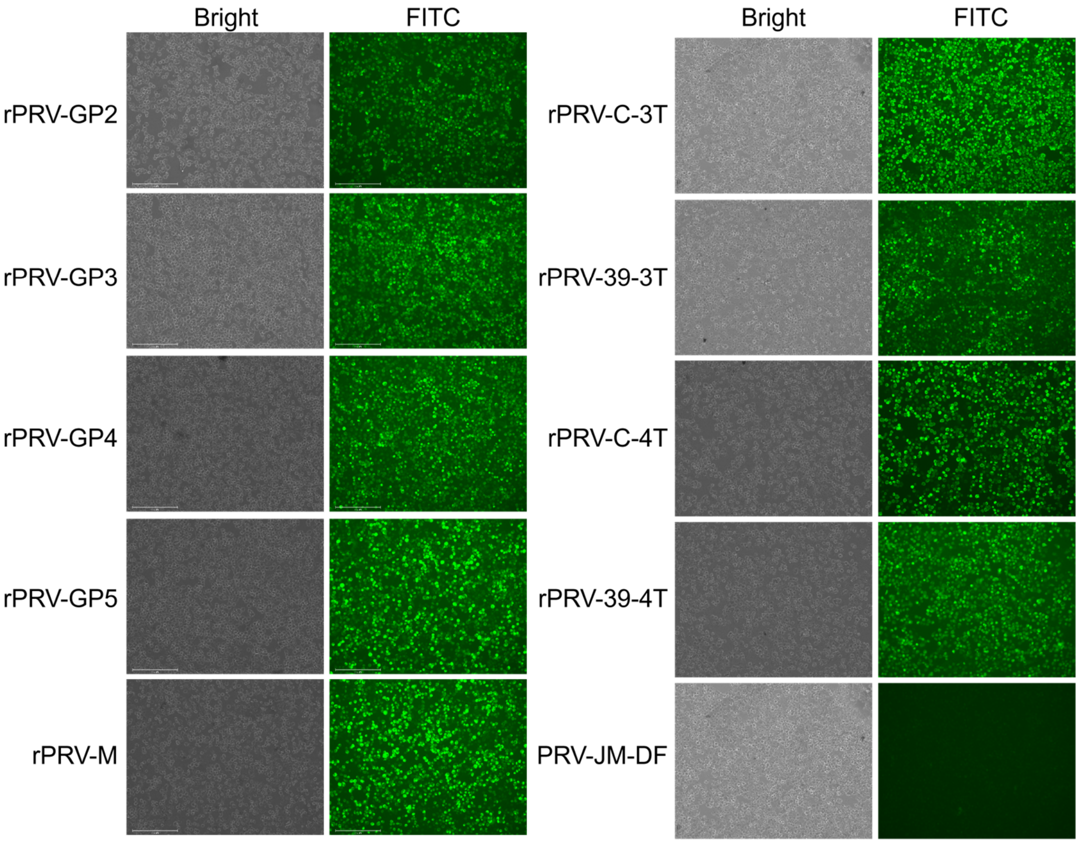

Figure S2. Identification of the expression of exogenous proteins of recombinant virus by IFA.

PK-15 cells in 12-well plate were infected with 500 $TCID_{50}$ of recombinant viruses for 48 h, and IFA was used to detect whether the exogenous proteins were expressed (the exogenous proteins are tagged with Flag, tested using Flag antibodies).

Figure S3

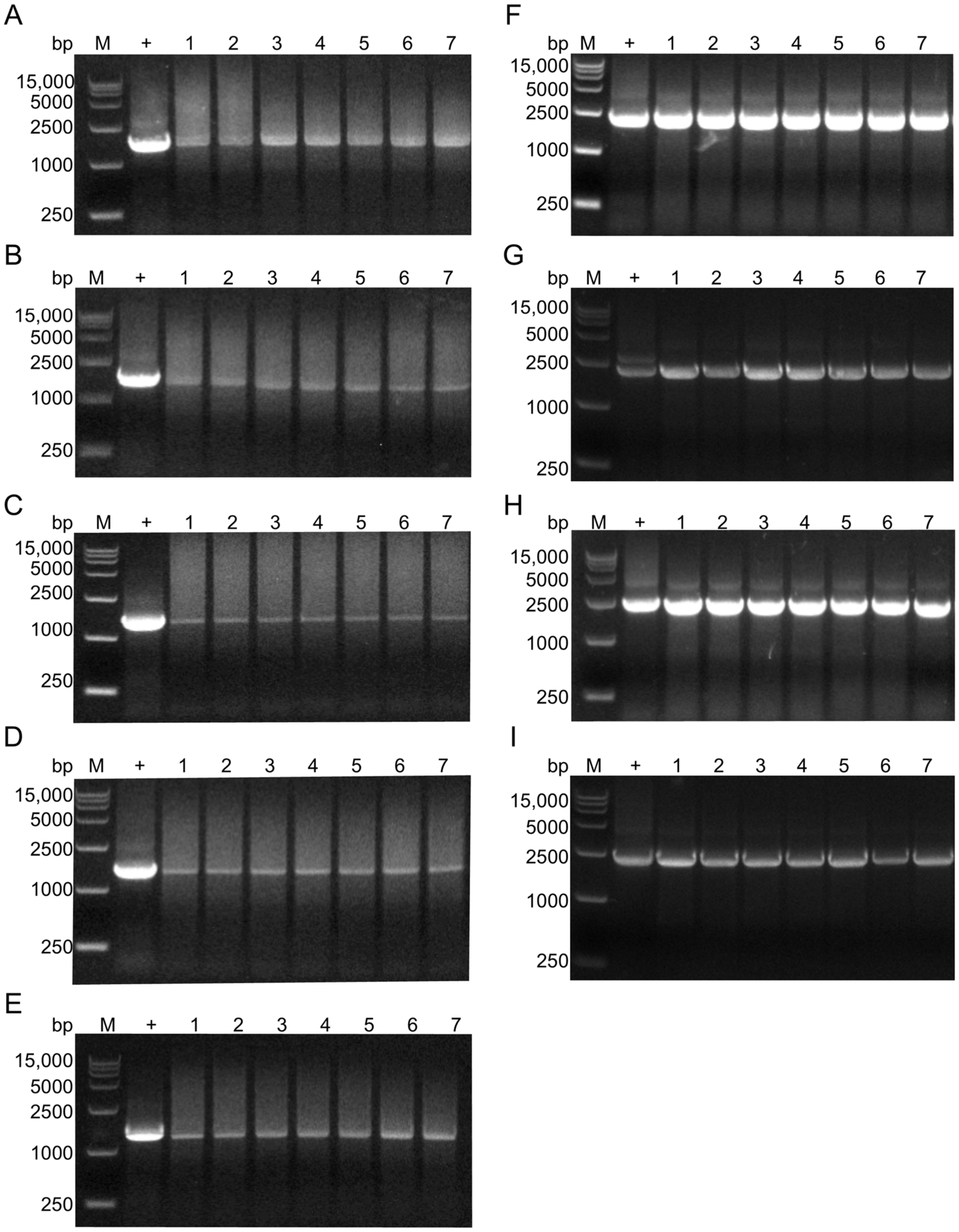

Figure S3. Identification of the genetic stability of recombinant viruses by PCR.

（A）rPRV-GP2；（B）rPRV-GP3；（C）rPRV-GP4；（D）rPRV-GP5；（E）rPRV-M；（F）rPRV-C-3T；（G）rPRV-39-3T；（H）rPRV-C-4T；（I）rPRV-39-4T；+: Positive control; Line 1: first generation viral genome; Line 2: fifth generation viral genome; Line 3: tenth generation viral genome; Line 4: fifteenth generation genome; Line 5: twentieth generation genome; Line 6: twenty-fifth generation genome; Line 7: thirtieth generation genome.

Figure S4

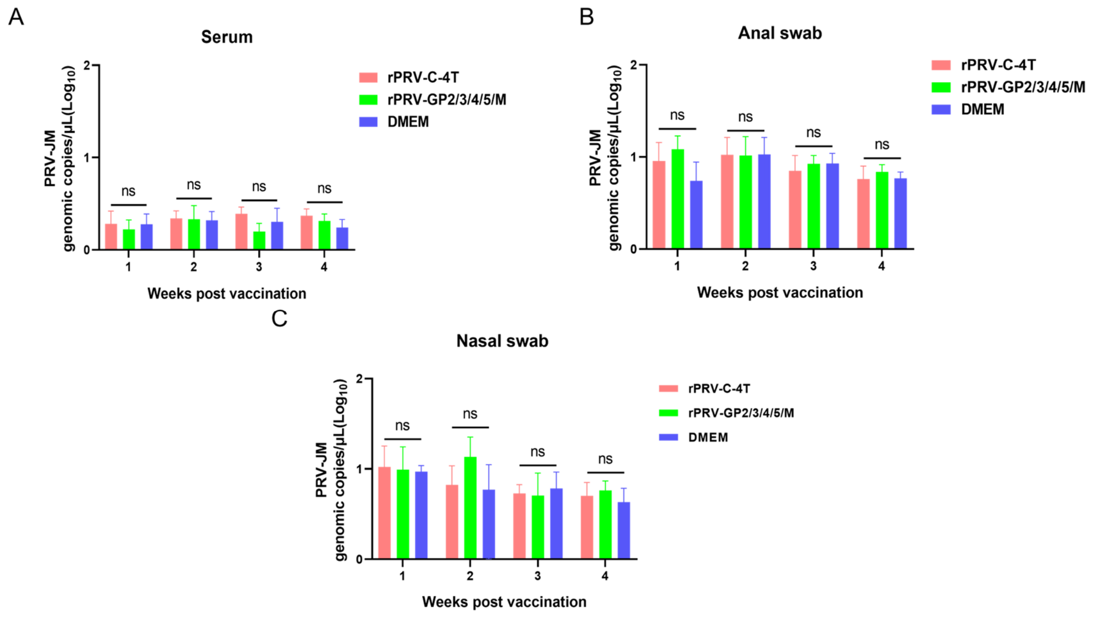

Figure S4. Virus load of PRV in piglet immune period.

The viral load of PRV in (A)serum, (B)anal swabs, and (C)nasal swabs four weeks after vaccination.

Figure S5

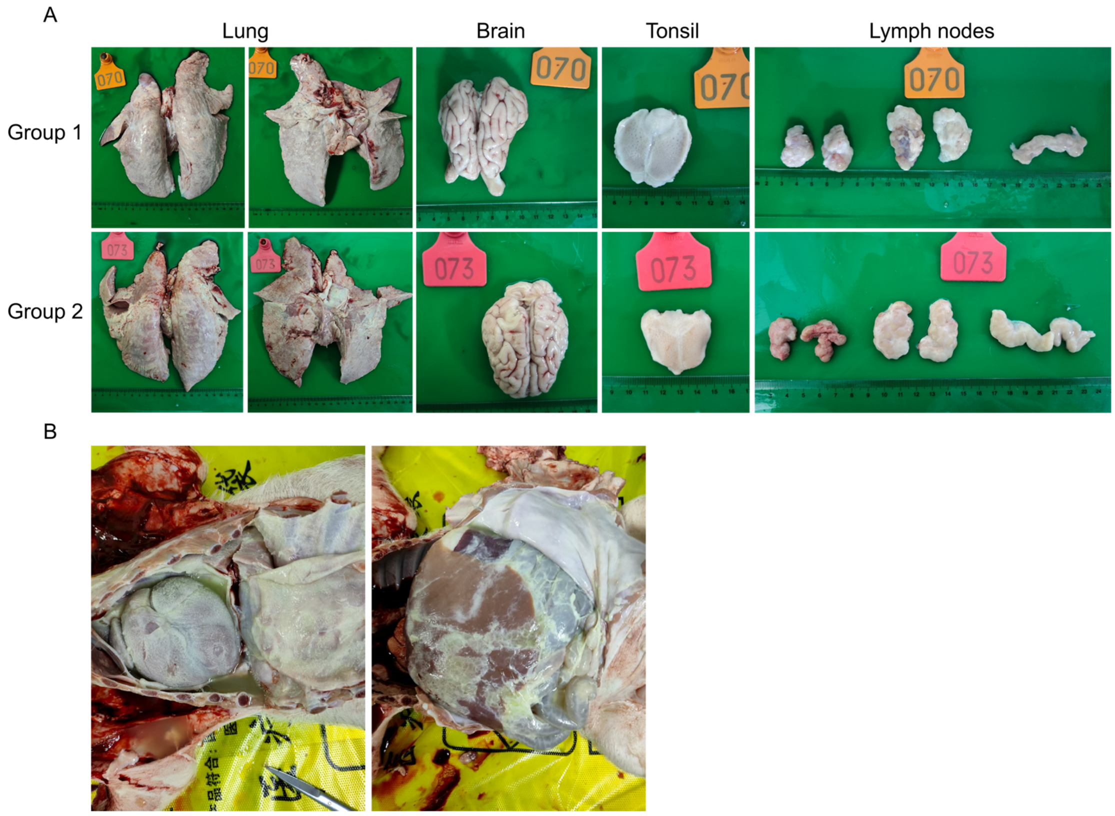

Figure S5. Tissue damage in piglets post-challenge.

(A) Group 1: The apparent pathological changes in surviving pigs of rPRV-GP2/3/4/5/M group; Group 2: The apparent pathological changes in pigs that died from secondary infection with Haemophilus parasuis in the PRRSV vaccine group; (B) The thoracic and abdominal cavities of pigs that died from secondary infection with Haemophilus parasuis in the PRRSV vaccine group.
