# Supplementary material for: Construction of Recombinant PRV Expressing PRRSV Antigens and Evaluation of Their Protective Efficacy in Pigs Against Homologous Challenge
Source: Vaccines (Basel). 2026 Oct 21;14(10):915. doi: 10.3390/vaccines14100915 (PMC13709669; doi:10.3390/vaccines14100915)
Supplement: Supplementary file 1 [file vaccines-14-00915-s001.zip › vaccines-4538747-Supplementary Tables.pdf]

Table S1. Primers used for constructing plasmids in this study.

Primer	Sequence（5’-3’）
TK-LA-F	GACCATGATTACGCCAAGCTTCGCTCTCGAGCGCCGAGTAGTG
TK-LA-R	GTAAGTTATGTAACGCCGTAGGCGCCGTCGAGGTAGATC
TK-RA-F	CATCAATGTATCTTATCGGAGATGGGGGTGTGACCCTC
TK-RA-R	CGTTGTAAAACGACGGCCAGTGAATTCAAGAAGAGCGCCTGCTG
gG-D- LA-F	GACCATGATTACGCCAAGCTTATGAAGTGGGCAACGTGGATCCTCGCC
gG-D- LA-R	GTAAGTTATGTAACGTCAGGCGGAGGCCACG
gG-D- RA-F	CATCAATGTATCTTAATGCTGCTCGCAGCGCTATTG
gG-D- RA-R	CGTTGTAAAACGACGGCCAGTGAATTCTACGGACCGGGCTGCG
UL11-10-LA-F	GACCATGATTACGCCAAGCTTAGGATGGCGGCGCTCGTTTTG
UL11-10-LA-R	GTAAGTTATGTAACGTCTAGAGACGGGAACGATGCGATAATATG
UL11-10-RA-F	CATCAATGTATCTTAGGATCCGGGCGGCGAGAGACGGACACGAGAG
UL11-10-RA-R	AAAACGACGGCCAGTGAATTCGGTATCGTGCGGGGACGGAAGC
EGFP-F	CGTTACATAACTTACGGTAAATGGCCCGCC
EGFP-R	TAAGATACATTGATGAGTTTGGACAAACCACAACTAGAATG
TKJD-F	GCCTTCTTAAGCGTCTCTCGGGGAAGCCCGAAGAG
TKJD-R	TCGTCGTTGCCTCCGTCGCGGTCTCCATC
CMV-F	GACATTGATTATTGACTAGTTATTAATAGTAATCAATTACGGGG
p39-F	GCGTACCTGTTCAACGACCCG
p39-R	CAATAACCAGCACGTTGCGCGGAGAGTCCCGA
TK-L-F	GACCATGATTACGCCAAGCTTCGCTCTCGAGCGCCGAGTAGTG
TK-L-R-CMV	TCAATAATCAATGTCCCGTAGGCGCCGTCGAGGTAGATC
TK-L-R-p39	GTTGAACAGGTACGCCCGTAGGCGCCGTCGAGGTAGATC
TK-R-F	TCGGAGATGGGGGTGTGACCCTC
TK-R-R	CGTTGTAAAACGACGGCCAGTGAATTCAAGAAGAGCGCCTGCTG
GP-F	ACGTGCTGGTTATTGTGCTGTC
3T-R	CACCCCCATCTCCGATCAGGGGCCAGGCTGGGCCTTC
4T-R	CACCCCCATCTCCGATCAAGGGCCTGGCTGAGCCTTC

Table S2. Primers used for sgRNA in this study.

Primer	Sequence（5’-3’）
EGFP sgRNA1-F	CACCGGGCGAGGAGCTGTTCACCG
EGFP sgRNA1-R	AAACCGGTGAACAGCTCCTCGCCC
EGFP sgRNA2-F	CACCTGGAGTTCGTGACCGCCGCC
EGFP sgRNA2-R	AAACGGCGGCGGTCACGAACTCCA
CMV sgRNA-F	CACCGGATAGCGGTTTGACTCACG
CMV sgRNA-R	AAACCGTGAGTCAAACCGCTATCC
TK sgRNA1-F	CACCCTCGACGGCGCCTACGGCAC
TK sgRNA1-R	AAACGTGCCGTAGGCGCCGTCGAG
TK sgRNA2-F	CACCGTGCGTGTGCGCCTTCACGT
TK sgRNA2-R	AAACACGTGAAGGCGCACACGCAC
gG-D sgRNA1-F	CACCGCGTACGGCCTTGCTTACGG
gG-D sgRNA1-R	AAACCCGTAAGCAAGGCCGTACGC
gG-D sgRNA2-F	CACCGGGGTGCGATCCACGCCCAG
gG-D sgRNA2-R	AAACCTGGGCGTGGATCGCACCCC
UL11-10 sgRNA1-F	CACCACGGGGGAGACGGAGGACGG
UL11-10 sgRNA1-R	AAACCCGTCCTCCGTCTCCCCCGT
UL11-10 sgRNA2-F	CACCGAGACGATGGCCGAGGACGG
UL11-10 sgRNA2-R	AAACCCGTCCTCGGCCATCGTCTC

Table S3. Primers used for qPCR in this study.

Primer	Sequence（5’-3’）
PRRSV-F	TTGTGCTTGCTAGGCCGC
PRRSV-R	ACGACAAATGCGTGGTTATCA
PRRSV-Probe	FAM-TCTGGCCCCTGCCCA-MGB
PRV-F	GAGGGGATCGCCGTGCTCTT
PRV-R	TCCGTGAAGCGGTTCGTGAT
PRV-Probe	FAM-CTACAAGAACGTCATCGTCACGACCG-MGB
